# Lipophilic prodrugs of nucleoside triphosphates as biochemical probes and potential antivirals

**DOI:** 10.1038/ncomms9716

**Published:** 2015-10-27

**Authors:** Tristan Gollnest, Thiago Dinis de Oliveira, Dominique Schols, Jan Balzarini, Chris Meier

**Affiliations:** 1 Institute of Organic Chemistry, Department of Chemistry, Faculty of Sciences, University of Hamburg, Martin-Luther-King-Platz 6, D-20146 Hamburg, Germany; 2 Department of Microbiology and Immunology, Laboratory of Virology and Chemotherapy, Rega Institute for Medical Research, KU Leuven, Minderbroedersstraat 10, B-3000 Leuven, Belgium

## Abstract

The antiviral activity of nucleoside reverse transcriptase inhibitors is often limited by ineffective phosphorylation. We report on a nucleoside triphosphate (NTP) prodrug approach in which the **γ**-phosphate of NTPs is bioreversibly modified. A series of Tri*PPP*ro-compounds bearing two lipophilic masking units at the **γ**-phosphate and d4T as a nucleoside analogue are synthesized. Successful delivery of d4TTP is demonstrated in human CD4^+^ T-lymphocyte cell extracts by an enzyme-triggered mechanism with high selectivity. In antiviral assays, the compounds are potent inhibitors of HIV-1 and HIV-2 in CD4^+^ T-cell (CEM) cultures. Highly lipophilic acyl residues lead to higher membrane permeability that results in intracellular delivery of phosphorylated metabolites in thymidine kinase-deficient CEM/TK^−^ cells with higher antiviral activity than the parent nucleoside.

## Abstract (web summary)

Charged phosphorylated metabolite such as nucleoside tri-phosphates exhibit poor membrane permeability due to their high polarity, limiting their utility as drugs or cellular probes. Here the authors develop a method to render nucleoside triphosphates cell permeable and allows their release by an enzyme-triggered mechanism.

## Graphical abstract

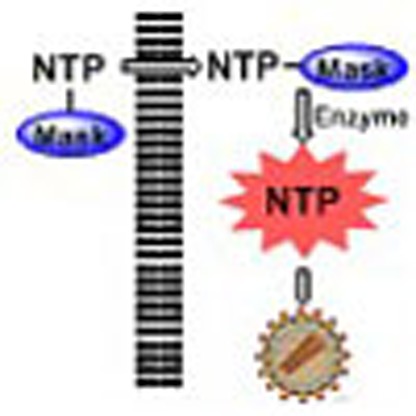

Over the last decades, a variety of nucleoside analogues were applied in antitumour and antiviral therapy and still play an important role to combat HIV, herpes virus, hepatitis B and hepatitis C virus infections^1^,^2^. The target of these nucleoside analogue drugs is the inhibition of the virus-encoded DNA polymerases, such as the HIV reverse transcriptase (RT)^3^,^4^ or the HCV-encoded RNA-dependent RNA-polymerase NS5B (ref. 5), which are the key enzymes in the replication cycle of HIV and HCV, respectively. To date, eight nucleoside analogues have been approved as HIV RT inhibitors (NRTIs)^6^. NRTIs are still used as the backbone of the combined antiretroviral therapy^7^. However, the antiviral efficacy of nucleoside analogues, such as the thymidine analogue 3′-deoxy-2′,3′-didehydrothymidine **1** (d4T) and 3′-deoxy-3′-azidothymidine (AZT), is dependent on the activity of host cell kinases metabolizing these nucleoside analogues into their antivirally active triphosphate forms (nucleoside triphosphates, NTPs)^8^,^9^,^10^,^11^.

The stepwise transformation via the nucleoside mono- (NMP) and diphosphates (NDP) into the corresponding NTP often occurs insufficiently because of the high substrate specificity of the involved kinases (Supplementary Fig. 1). Furthermore, many nucleoside analogues have limitations such as poor biological half-lives, variable bioavailability after oral administration or selection of drug resistance, which reduce their clinical efficacy^12^,^13^. To overcome these hurdles, the usage of prodrugs of the phosphorylated metabolites have been explored in the past^14^,^15^.

In the case of d4T **1**, the first phosphorylation step to yield its monophosphate form **2** catalysed by the host cell enzyme thymidine kinase (TK) is metabolism-limiting because of the rather modest affinity of d4T **1** to TK as an alternative substrate and because TK activity is S-phase-dependent^11^,^16^,^17^. However, to avoid this limitation, it is not possible to apply the charged monophosphorylated metabolite because of the high polarity and thereby extremely poor, if any, membrane permeability. The development of nucleotide prodrugs capable of delivering the monophosphorylated metabolite and thereby bypassing the intracellular activation offered advantages over the use of the corresponding nucleoside analogue^18^,^19^. Moreover, lipophilic-masked NMPs such as **3** are less vulnerable to degradation by unspecific phosphatases present in the blood. This enhanced not only the plasma half-life but also enables the prodrug to be taken up by cells through passive diffusion^20^,^21^. A number of successful NMP prodrug strategies were reported in the past that efficiently bypass the nucleoside kinase hurdle. These prodrug forms such as phosphoramidates and *cyclo*Sal-phosphate triesters of nucleoside analogues were indeed shown to deliver the NMP either by chemical or enzymatic hydrolysis in the target cells^22^,^23^,^24^,^25^,^26^,^27^. In addition to NMPs also the successful delivery of acyclic nucleoside phosphonates such as cidofovir has been reported^28^. However, all these approaches delivered the monophosphor(n)ylated forms of the nucleosides that subsequently needed further phosphorylation into the triphosphate forms by cellular kinases to inhibit their target polymerase.

However, not in all cases such NMP prodrug strategies were successful. For instance, in the case of AZT the metabolism is limited by the second conversion step, the formation of AZT-diphosphate by thymidylate kinase^10^,^29^. In this case, a lipophilic prodrug that intracellularly releases the NDP would be desirable. At the same time, this would avoid toxicity caused by the parent nucleoside or the accumulation of the monophosphate form^30^. A further example is 2′,3′-dideoxy-2′,3′-didehydrouridine (d4U). The parent nucleoside proved to be completely inactive against HIV replication in cell cultures. In contrast, the triphosphate form of d4U is one of the most effective inhibitors of the HIV's RT^31^.

We reported on NDP prodrugs (Di*PP*ro-approach), which selectively released NDPs not only in phosphate buffer (pH 7.3) but for the first time also in CEM cell extracts^32^,^33^,^34^,^35^. These compounds showed very good antiviral activity in TK-deficient CEM/TK^−^ cell cultures infected with HIV, proving the uptake of the compounds and the delivery of at least phosphorylated metabolites, most likely the NDP. The uptake of those compounds was achieved by two acceptor-substituted benzylesters linked to the β-phosphate group of the NDP. The stability of the compounds correlated with the length of the aliphatic residue of the mask^32^,^33^. However, we have shown that the delivery of the corresponding mono- or diphosphates from prodrug forms (such as the *cyclo*Sal- or Di*PP*ro-compounds) did not improve the antiviral activity^34^. Importantly, in the same study we have proven that d4U diphosphate was a very poor substrate for the NDP kinase, the enzyme that is generally accepted to be involved in the conversion of NDPs into their triphosphate forms. Thus, this study showed that the phosphorylation of nucleotide analogues by NDP kinase can be also rate-limiting in the activation process of a nucleoside analogue^34^,^36^. As a consequence, the development of nucleoside triphosphate prodrugs would be highly interesting and desirable because this would bypass *all* steps of intracellular phosphorylation and would maximize the intracellular concentration of the ultimately bioactive NTP. Although this has been recognized before^11^, it was also claimed that the development of prodrugs of NTP is chemically not feasible because of the low stability of such compounds^37^. For this reason, NTPs were very rarely used as drug platforms because of their expected poor deliverability and their high sensitivity for enzymatic dephosphorylation. Thus, very few reports on potential triphosphate prodrugs have been reported^38^,^39^. In addition, in the few reported examples the yields in the chemical synthesis were poor, and additionally the compounds proved hydrolytically very unstable. A difficulty that has to be taken into account in the development of NTP prodrugs is related to the energy-rich phosphate anhydride bonds within the triphosphate unit. Under physiological conditions, these linkages are only kinetically stable because of the charges present at that moiety, which prevent nucleophilic reactions that end up in the cleavage of these anhydride bonds but can be enzymatically cleaved. Interestingly, **γ**-modification of NTPs by esterification or replacement of the **γ**-phosphate group by a phosphonate moiety led to a marked increase in enzymatic stability of the triphosphate unit^40^,^41^. On the other hand, complete lipophilic modification and thereby neutralization of the charges would significantly increase the reactivity of the triphosphate unit. For completeness, it should be added that the delivery challenge for NTPs has also been addressed by formulating these compounds with cationic nanogels. However, this approach still requires elaboration with respect to their toxicity, immunogenicity and pharmacokinetics^42^,^43^.

Here we disclose the development of a novel prodrug concept for NTPs that releases directly NTPs with high selectivity by an enzyme-triggered mechanism and thus allows the bypass of *all* phosphorylation steps normally needed for the activation of a nucleoside analogue (Supplementary Fig. 2). To achieve this goal, the γ-phosphate group of a NTP was modified by esterification with acyloxybenzyl moieties (Tri*PPP*ro-compounds). By fine-tuning the lipophilicity through the use of different acyl esters the chemical stability also proved controllable so that the polarity caused by the remaining charges at the α- and β-phosphates could be efficiently compensated.

The cleavage of the prodrug moieties is initiated by an enzymatic hydrolysis of the phenolic acyl-ester. This reaction leads to a spontaneous cleavage of the benzyl C–O bond forming first a monomasked intermediate of the NTPs. This process is repeated for the second mask so that finally the NTP is formed^32^. Lipophilic aliphatic esters have proven to be suitable for prodrugs to allow entering the cell independently of nucleoside transporters but to enable removal of the lipophilic prodrug moieties by cellular esterases/lipases^44^. Moreover, fatty-acid esters are known to be taken up by the mononuclear phagocyte system. These cells are important in the pathogenesis of AIDS and are considered to be reservoirs for HIV particles^45^,^46^.

We report on the synthesis of NTP prodrugs **3** bearing two identical 4-alkanoyloxybenzyl- (**a**–**k**), 4-alkoxycarbonyloxybenzyl- (**l–n**) and 4-aminocarbonyloxybenzyl groups (**o**–**q**), their hydrolysis properties in different media, the hydrolysis mechanism, primer extension assays and their anti-HIV activity. In addition, the synthesis of the monomasked (4-alkyloxybenzyl)-d4TTPs **4a**,**e**,**j** is reported. As a model nucleoside analogue, d4T **1** was used to allow a comparison of the Tri*PPP*ro-compounds **3** with the Di*PP*ro-compounds.

## Results

### Synthesis of Tri*PPPr*o-d4T triphosphate prodrugs 4

**Figure 1:**
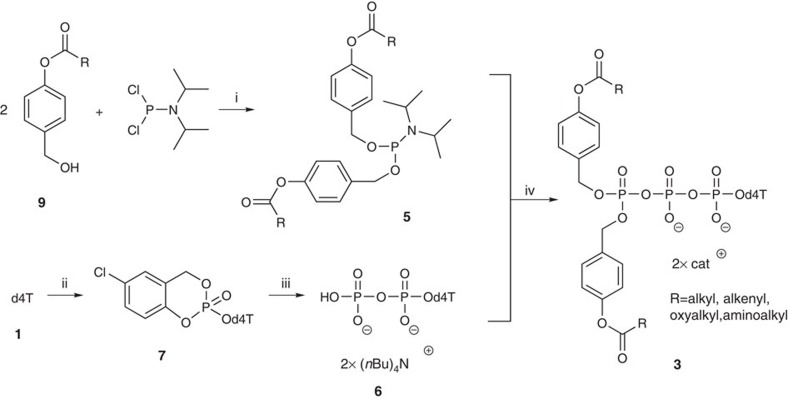
Reagents and conditions. (i) Triethylamine, THF, 0 °C-rt, 20 h; (ii) **1.** 5-chlorosaligenylchlorophosphite **8**, *N,N*-di*iso*propylethylamine, CH_3_CN, −20 °C-rt, 3 h, **2**. *t*-BuOOH in *n*-decane, 0 °C-rt, 30 min; (iii) (H_2_PO_4_)Bu_4_N, DMF, rt, 20 h; (iv) **1**. DCI, CH_3_CN, rt, 1 min, **2**. *t*-BuOOH in *n*-decane, 0 °C-rt, 15 min.

For the synthesis of Tri*PPP*ros-d4TTP prodrugs **3** a convergent strategy using a dicyanoimidazol (DCI)-mediated coupling of an appropriate phosphoramidite **5** and d4TDP **6** to form the energetically rich pyrophosphate moiety in the last step was performed (Fig. 1). First, d4T **1** was prepared in good overall yields according to a three-step protocol reported by *Horwitz*^47^. From that compound, d4TDP **6** was prepared by applying the *cyclo*Sal technique (55% yield) because it has been reported that acceptor-substituted *cyclo*Sal nucleotides gave access to diphosphorylated compounds by using tetra-*n*-butylammonium phosphate as a nucleophile^48^. Therefore, the 5-chloro-substituted *cyclo*Sal-phosphate triester **7** was synthesized starting from d4T **1** with 5-chlorosaligenylchlorophosphite **8** followed by oxidation with *tert*-butylhydroperoxide to give the product **7** as a mixture of two diastereomers in high yields.

Despite its very difficult chromatographic properties, the resulting crude (*n*-Bu)_4_N^+^-salt **6** was purified by automatic RP-18 flash chromatography without additional ion exchange. The hygroscopic tetra-*n*-butylammonium salt form of d4TDP **6** was co-evaporated in dimethylformamide (DMF) and dried in high vacuum before the coupling reaction to ensure dry reaction conditions. The use of tetra-*n*-butylammonium counterions afforded a higher reactivity and better solubility in organic solvents. D4TDP **6** was then reacted with a series of phosphoramidites **5** in a very fast DCI-mediated coupling reaction and was oxidized^32^,^33^.

**Figure 2:**
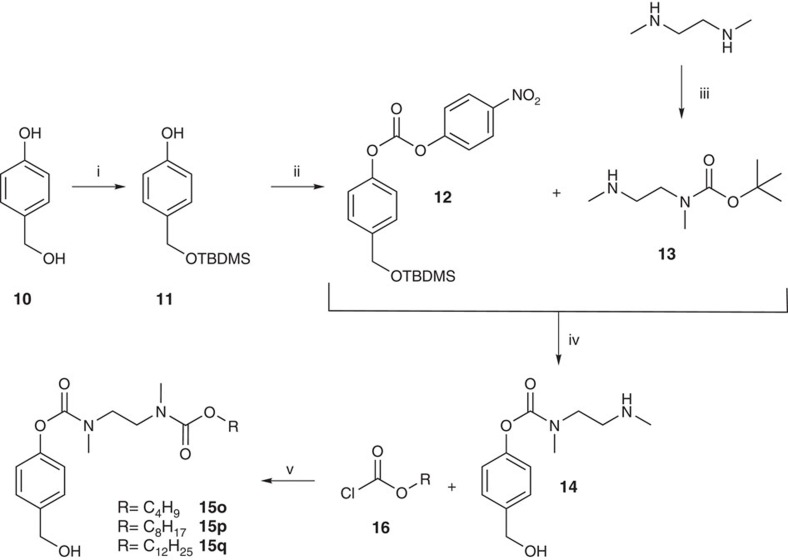
Reagents and conditions. (i) TBDMSCl, imidazole, CH_2_Cl_2_, rt, 2 h; (ii) **1**. 4-nitrophenyl chloroformiate, triethylamine, CH_2_Cl_2_, rt, 16 h, **2**. triethylene glycol monomethyl ether, rt, 20 min; (iii) Boc_2_O, 0 °C-rt, 20 h; (iv) **1**. 4-DMAP, di*iso*propylethylamine, toluene, rt, 16 h, **2**. TFA/CH_2_Cl_2_, rt, 0.5 h; (v) **1**. TMSCl, imidazole, THF, 0 °C-rt, 2 h, **2**. triethylamine, 0 °C-rt, 1.5 h, **3**. 1% HCl (12 M) in EtOH, rt, 1 h.

Inspired by recently published biodegradable linear polymers that were degraded by an acid-induced cascade reaction, we also synthesized carbamate derivatives **3o**–**q** (ref. 49). In this case, 4-hydroxybenzyl alcohol **10** was first protected with *tert*-butyldimethylsilyl chloride to give compound **11** followed by an esterification using 4-nitrophenyl chloroformiate. To trap the excess of the chloroformiate, triethylene glycol monomethyl ether was added^49^. The obtained carbonate **12** was then converted with *t*-Boc-protected dimethylethylenediamine **13** and after acid-catalysed cleavage of both protecting groups the methylcarbamate **14** was isolated in 98% yield. Finally, phenylcarbamates **15** were synthesized starting from **14** in an one-pot reaction including tetramethylsilane (TMS) protection, coupling with the corresponding acyl chloroformiates **16** and desilylation in a yield of up to 59% (Fig. 2).

The 4-hydroxybenzyl alcohols bearing acyl-ester groups at the phenol and the carbamates were converted into phosphoramidites **5** in high yields as published before^32^,^33^. Finally, d4TDP **6** was mixed with 1.7 equivalents (eq.) of a corresponding phosphoramidite **5** and co-evaporated with acetonitrile. Then, the mixture was dissolved in a minimum of acetonitrile because achieving a high concentration was crucial for the success of the coupling reaction. In case of compounds with long acyl residues (R≥C_11_H_23_), tetrahydrofurane (THF) was added to accomplish complete solubility of the reagents. In some cases, the conversion of d4TDP **6** was not complete. In these cases, all volatile components were removed *in vacuum*, the residue was redissolved and further 1.0 eq. of the phosphoramidite **5** and 0.8 eq. of DCI were added. After another minute of stirring, the reaction mixture was oxidized. After oxidation the quantitative consumption of d4TDP **6** was confirmed using high-performance liquid chromatography (HPLC; Supplementary Fig. 3). Next, the solvent was removed *in vacuum* and the crude product was purified with RP-18 chromatography using gradients of water/acetonitrile or water/THF as eluents. For compounds **3a**–**f**, **l**–**n**, **o**–**q** the tetra-*n*-butylammonium ions were exchanged by ammonium ions using Dowex 50WX8 and the chromatography was repeated.

### Synthesis of monomasked triphosphate prodrugs 4

**Figure 3:**
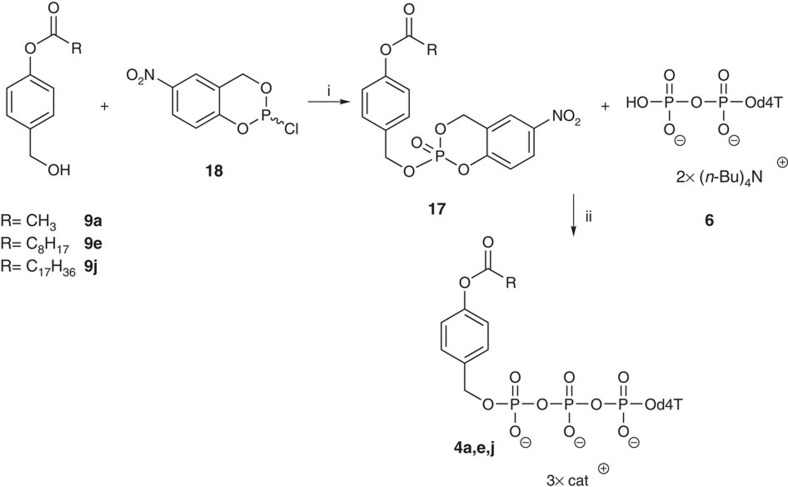
Reagents and conditions. (i) **1**. di*iso*propylethylamine, CH_3_CN, −20 °C-rt, 1 h, **2**. oxone, H_2_O/CH_3_CN, rt, 30 min; (ii) bis(tetra-*n*-butyl)ammonium-d4TDP **6**, DMF, rt, 3 h.

In addition to the Tri*PPP*ro-compounds **3**, the monomasked acyloxybenzyl-NTP derivatives **4** were synthesized as well (Fig. 3). Such monoesterified substances were described by others as potential triphosphate prodrugs^38^,^39^,^50^. Several synthesis routes mainly based on DCC-activated coupling were published. In our recent studies with Di*PP*ro-prodrugs, we isolated such monoesterified compounds by simple hydrolysis^33^.

An efficient access to such compounds was developed in this study. The monoesterified NTPs were prepared starting from 4-acyloxybenzyl alcohol **9** and its conversion into the 5-nitro-*cyclo*Sal-triester **17**. Despite the high reactivity of this compound, the purification by preparative thin-layer chromatography (TLC) was successful. The benzyl-(5-nitro-*cyclo*Sal)-phosphate triesters **17** were obtained in high yields (up to 89%). Next, triesters **17** gave the monomasked acyloxybenzyl-d4TTPs **4** in yields of 26–30% by the addition of d4TDP **6**.

### Stability studies

The Tri*PPP*ros-d4TTP prodrugs **3** and the intermediates **4** were incubated in PBS (25 mM, pH 7.3), or were exposed to pig liver esterase (PLE) in PBS and to human CD_4_^+^ T-lymphocyte cell extracts to study their stability and the product distribution. The hydrolysis mixtures were analysed by means of analytical RP-18-HPLC. The calculated half-lives (Table 1) were determined for the first removal of one masking unit (*t*_1/2_(1)) to yield the intermediate **4** and the second hydrolysis step (*t*_1/2_(2)) to give the triphosphate **19**.

### Chemical stability in PBS at pH 7.3

In PBS, the stability of Tri*PPP*ro-d4TTP prodrugs **3a**–**h**,**l**–**n** increased with increasing alkyl chain lengths (Table 1). However, the half-lives of more lipophilic compounds **3i**–**k** decreased because of altered solubility behaviour or micelle formation. The half-lives of the intermediates **4** were always considerably higher than those of their precursors **3** because of the increase in charges leading to repulsive interaction with an incoming nucleophile. Moreover, formation of the three nucleotide forms **2**, **6** and **19** were observed (Supplementary Fig. 4).

### Hydrolysis study using esterase

Next, we examined the enzymatic stability of prodrugs **3a**–**n** by incubation with PLE in PBS, pH 7.3. The cleavage of the masking units for **3b**–**g** occurred much faster than that in PBS, demonstrating a significant contribution of the enzymatic cleavage (Table 1). As observed in the chemical hydrolysis studies, the cleavage of the second masking group proceeded much slower. According to the substrate specificity of PLE we determined the lowest half-lives for **3c**–**f** and **3m**. Shorter as well as longer alkyl residues in the ester moiety of the masking group led to increased half-lives. In addition, d4TTP **19** was delivered by enzymatic activation of **3a**–**n** but was also found to be the sole metabolite from **4a**,**e**,**j** as long as the enzymatic cleavage occurred rapidly (Supplementary Fig. 5). The carbamate-functionalized prodrugs **3o**–**q** were not substrates for PLE, as expected.

**Figure 4:**
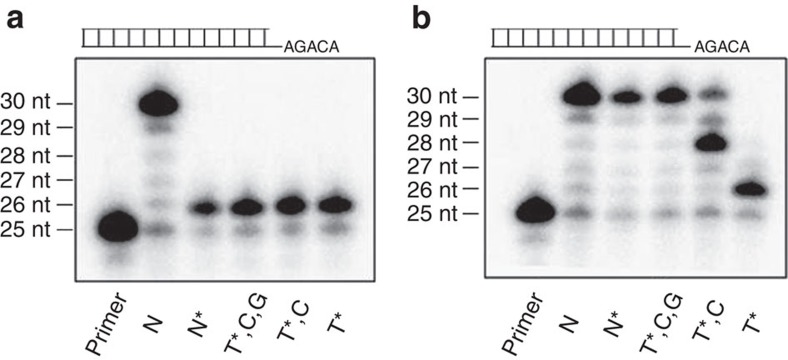
Primer extension assays with HIV reverse transcriptase. (**a**) PLE hydrolysate based on Tri*PPP*ro-d4TTP **3e** (T*); dCTP; dGTP; all natural triphosphates (N); dATP, dCTP, dGTP and the hydrolysate of **3e** (N*). (**b**) PLE hydrolysate based on Tri*PPP*ro-compound **3r** (T*); dCTP; dGTP; all natural triphosphates (N); dATP, dCTP, dGTP and the hydrolysate of **3r** (N*). nt: nucleotide, length of primer.

**Figure 5:**
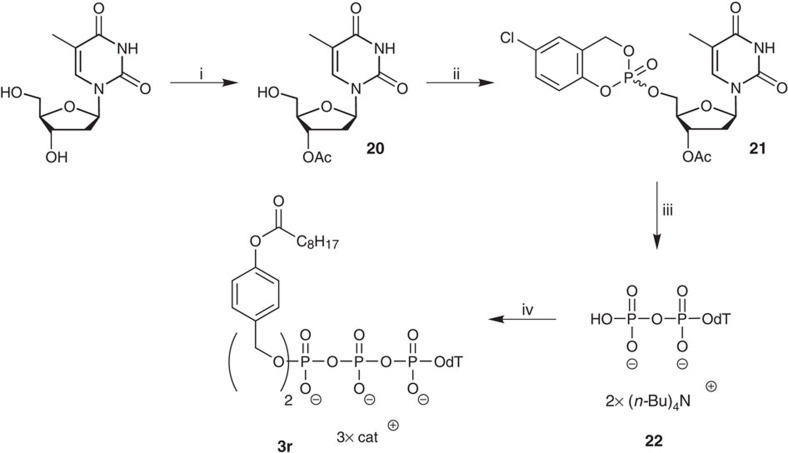
Reagents and conditions. (i) **1**. TBDMSCl, pyridine, rt, 20 h, **2**. Ac_2_O, rt, 5 h, **3**. TBAF, THF, 0 °C, 1.5 h; (ii) **1**. 5-chlorosaligenylchlorophosphite **8**, *N,N*-di*iso*propylethylamine, CH_3_CN, 0 °C-rt, 3 h, **2**. *t*-BuOOH in *n*-decane, 0 °C, 20 min; (iii) **1**. (H_2_PO_4_)Bu_4_N, DMF, rt, 20 h, **2**. MeOH/H_2_O/Bu_4_NOH (7:3:1), rt, 17 h; (iv) **1**. **5e**, DCI, CH_3_CN, rt, 1 min; **2**. *t*-BuOOH in *n*-decane, 0 °C-rt, 15 min.

To confirm the prodrug concept and thus the direct successful release of the biologically active triphosphate metabolite, the prodrug **3e** was exposed to PLE and the hydrolysis monitored with RP-18-HPLC. After complete consumption of **3e** as well as its intermediate **4e** the solvents were removed. For the template/primer extension assay (Fig. 4a), HIV RT was incubated with the PLE hydrolysate as such (T*) or with the PLE hydrolysate in the additional presence of dCTP (T*, C), or dCTP+dGTP (T*, C, G) or all natural 2′-deoxynucleotides (N*). Interestingly, an immediate DNA chain termination was observed after incorporation of d4TMP **2** (derived from the incoming d4TTP **19** that was released from the prodrug by PLE), while the control reaction containing all four natural NTPs in the absence of the PLE hydrolysate (N) showed full extension of the primer (Fig. 4a). In addition, Tri*PPP*ro-TTP **3r** was synthesized (Fig. 5) and also investigated in the same way. As expected, the template/primer extension assay showed efficient DNA elongation (Fig. 4b). The T* lysate resulted in termination of the polymerization because of the lack of the next complementary nucleotide (dCTP; position 26 nt), whereas the reaction proceeded till position 28 nt in the presence of both T* and dCTP (T*, C). The primer could be fully extended till 30 nt when T* was added in the presence of dCTP and dGTP, as also the N* and N samples could.

### Hydrolysis in cell extracts

The hydrolysis of the Tri*PPP*ro-compounds **3** was further investigated in human CD_4_^+^ T-lymphocyte CEM cell extracts. Again, the half-lives of the prodrugs **3a**–**n** correlated well with chain length and were significantly lower than the half-lives in PBS buffer (Table 1). Thus, an enzymatic cleavage reaction took place as described above for the PLE studies. Furthermore, we observed the formation of the corresponding intermediates **4a**–**n** that had lower half-lives than their parent prodrugs. This assumption was proven by hydrolysis of the synthesized intermediates **4a**,**e**,**j**. In contrast to hydrolysis studies with PLE, in addition to d4TTP **19** d4TDP **6** was also detected as a major component in the CEM cell extracts most probably because of the presence of hydrolytic enzymes such as phosphatases and esterases (Supplementary Fig. 6).

The formation of d4TDP **6** was clearly not a result of unselective cleavage of the prodrug **3**. Investigations in cell extracts starting from d4TTP **19** led to a rapid degradation (*t*_1/2_=0.63 h). For this reason d4TDP **6** (*t*_1/2_=59 h) accumulated under these conditions. On the other hand, only very small amounts of d4TMP **2** were formed. Nevertheless, it was proven that the triphosphate of d4T **19** was successfully released in biological media such as CD4^+^ T-lymphocyte extracts.

### Antiviral evaluation

Tri*PPP*ro-compounds **3a**–**n** were evaluated for their ability to inhibit the replication of HIV. For this purpose, HIV-1- or HIV-2-infected wild-type CEM/0 as well as mutant TK-deficient CEM cell cultures (CEM/TK^−^) were treated with the prodrugs **3**. As can be seen in Table 1, all compounds showed virtually similar activities against HIV-1 and HIV-2 as the parent nucleoside d4T **1**. A somewhat increased antiviral activity with increasing lipophilicity resulting from their advantageous permeability was observed. In addition, all prodrugs with R≥C_8_H_17_ were also highly potent in CEM/TK^−^ cells, whereas d4T **1** lacked relevant anti-HIV activity as expected in this TK-deficient cell model (EC_50_:150 μM). It should also be noticed that none of the prodrugs **3** were endowed with a significantly higher cytotoxicity than the parent d4T **1** compound (Table 2).

## Discussion

We reported on the first successful direct intracellular delivery of NTPs using prodrug technology. D4T triphosphate prodrugs **3a**–**q** were prepared via a convergent route using phosphoramidite chemistry (Fig. 1). Despite complete and selective conversion, Tri*PPP*ro-NTP prodrugs **3** were obtained in yields between 27 and 66%. We assumed that the loss in yield may be the result of a cleavage of the β- and γ-phosphate anhydride bond of the prodrugs during work-up. This assumption was supported by the detection of d4TDP **6** and the bis(benzyl)phosphate diester after chromatography. Alternative purification methods such as extraction and precipitation were investigated but proved to be inefficient. Nevertheless, by this method a large number of Tri*PPP*ro-d4TTPs **3** bearing various acyloxybenzyl-masking units were obtained. Moreover, this synthesis strategy also showed to be applicable to the synthesis of Tri*PPP*ro-compounds bearing other pyrimidine or purine nucleoside analogues. In addition, the intermediates **4a**,**e**,**j** were synthesized using the *cyclo*Sal method and were obtained in moderate chemical yields. This method, which is based on the *cyclo*Sal strategy, is a reliable method for the synthesis of polyphosphate diesters comprising esters at both ends of the polyphosphate group (Fig. 3)^48^,^51^,^52^.

**Figure 6:**
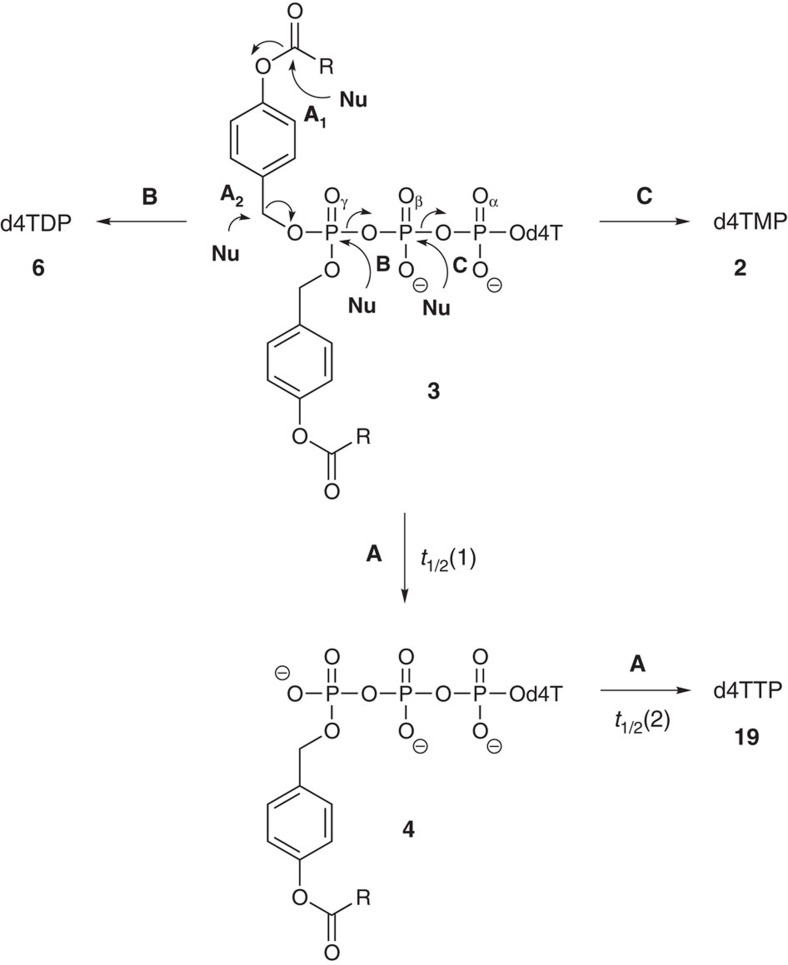
Chemical hydrolysis pathways for Tri*PPP*ro-d4TTPs **3**. A_1+2_ delivery of d4TTP **19**; B release of d4TDP **6** by cleavage of the phosphoanhydride bond between β- and γ-phosphate; C release of d4TMP **2** by cleavage of phosphoanhydride bond between α- and β-phosphate.

In general, three reactions should be considered in the hydrolysis pathways of Tri*PPP*ro-nucleotide prodrugs **3**. First, the designed pathway yielding the NTP; second, a concurrent reaction that involved a nucleophilic reaction at the γ-phosphate leading to the formation of d4TDP **6**; and third, a nucleophilic reaction at the β-phosphate that would lead to d4TMP **2**. Figure 6 summarizes all three possible hydrolysis pathways leading to the different phosphorylated nucleotide species. As shown in Fig. 6, to release d4TTP **19** two successive cleavage processes were necessary (path A). Thus, in addition to hydrolysis pathway **A**, also a reaction at the γ- and β-phosphate groups took place as side-reactions. Owing to the presence of a second energetically rich pyrophosphate bond, but despite its additional negative charge, the half-lives were found to be lower than those published recently for the Di*PP*ro-d4TDPs^33^. However, the triphosphate **19** was the predominant product formed (Supplementary Fig. 4). Moreover, after the starting material **3e** was completely consumed, there was no further increase in the amounts of d4TMP **2** and d4TDP **6**, which again points to the fact that the intermediate selectively delivers the triphosphate while the mono- and the diphosphate are formed from the starting Tri*PPP*ro-compounds only. A comparable behaviour has also been observed for the Di*PP*ro-compounds^33^.

In contrast, for the carbamate derivatives **3o**–**q** the cleavage of the masking groups occurred only once leading to the intermediates **4o**-**q**. Therefore, it was concluded that the delivery mechanism should be different as compared with the ester-bearing masking groups. Owing to the highly stable carbamate functions present in the masking group, the first masking group cannot be cleaved by the original mechanism that involves a cleavage within the ester/carbamate residue. To gain more insights into this, a hydrolysis experiment of derivative **3o** was conducted in ^18^O-labelled water to yield **4o**. Surprisingly, the ^18^O-label was found in the cleaved benzyl-alcohol and not at the phosphate, which was convincingly confirmed using mass spectrometry (Supplementary Fig. 7). Two different interpretations are possible for this result: (i) a S_N_1-type reaction took place forming a benzyl cation and the monomasked NTP intermediate **4**. The cation is then trapped by addition of water or (ii) a S_N_2-type reaction took place instead in which the labelled water displaces the monomasked NTP intermediate **4** (Fig. 6, hydrolysis pathway **A**_**2**_). In addition to hydrolysis in PBS, pH 7.3, **4p** was hydrolysed under slightly acidic conditions (pH 6.0), although in comparison with the physiological pH conditions no difference in its hydrolysis behaviour was observed. Because of the very long hydrolysis time periods, a cleavage of the glycosidic bond in d4T **1** resulted in the appearance of the nucleobase thymine. The amount doubles every 63 h; however, this aspect has not been further considered in these investigations because it was irrelevant in the enzyme or cell extract incubations and in the case of other nucleoside analogues.

Finally, a very important result from the studies conducted with the monomasked intermediates **4a**,**e**,**j** was the finding that exclusively d4TTP **19** was formed from these compounds. In addition, the hydrolysis studies conducted in the presence of PLE clearly led to the selective formation of two different NTPs (d4TTP and TTP) from the corresponding prodrug forms.

In conclusion, because of a successful cell membrane passage of the Tri*PPP*ro-compounds **3** and subsequent intracellular enzymatic hydrolysis, which led to the direct intracellular formation of phosphorylated d4T metabolites such as d4TTP **19** or at least d4TDP **6**, marked anti-HIV activity in CEM/TK^−^ cell cultures was observed while the parent nucleoside d4T **1** lacked significant activity in this cell assay. Thus, although the Tri*PPP*ro-compounds are still charged at the phosphate groups, obviously the modification at the γ-phosphate group by lipophilic, bioreversible moieties gives the molecule sufficient lipophilicity to penetrate the cell membrane. To the best of our knowledge, we provided the first direct proof of the successful application of masked triphosphates that obviously are able to efficiently enter the cells and to directly deliver a higher phosphate derivative, most likely d4TTP **19**. Because the concept should be generally applicable to natural nucleosides and a broad variety of nucleoside analogues, a novel way to deliver the corresponding bioactive triphosphate form of these nucleosides without any need for further enzymatically catalysed phosphorylation has been discovered. This concept seems to be very interesting for application with nucleoside analogues that show severe limitations in their activation to give the corresponding NTPs. Moreover, we are convinced that this approach is not limited to HIV treatment but can also be used for other viral targets and cancer and can also be used as a delivery for non-natural NTPs as biochemical tools in Chemical Biology approaches.

## Methods

### General

All reactions were carried out under dry conditions and at room temperature. *Solvents and reagents*: Acetonitrile, THF and DMF were purchased from Acros Organics (Extra Dry over molecular sieves) and dried with activated molecular sieves. Triethylamine and *N,N*-di*iso*propylethylamine were refluxed over CaH_2_ for 3 days and distilled under nitrogen. 5-Chlorosaligenylchlorophosphite **8** and 5-nitrosaligenylchlorophosphite **18** were synthesized according to the literature and **8** freshly distilled before use^48^. All further reagents commercially available were used as received. *Thin-layer chromatography*: For TLC Macherey–Nagel pre-coated TLC sheets Alugram Xtra SIL G/UV254 were used; sugar-containing compounds were visualized with sugar spray reagent (4-methoxybenzaldehyde/EtOH/concentrated sulphuric acid/glacial acetic acid in ratio 5/90/5/0.1 v/v) and phosphate-containing compounds with ammonium molybdate solution (1 g (NH_4_)_6_Mo_7_O_24_ 4 H_2_O in 7 ml semiconcentrated nitric acid and 13 ml water) followed by tin(II)chloride solution (0.1 g SnCl_2_ 2 H_2_O in 20 ml 0.5 mol l^−1^ hydrochloric acid). *Preparative chromatography*: The preparative TLCs were accomplished with a chromatotron (Harrison Research, Model 7,924T) using glass plates coated with 2 or 4 mm layers of Merck 60 PF_254_ silica gel. *Column chromatography*: Normal phase column chromatography was performed with Macherey–Nagel silica gel 60 M (0.04–0.063 mm). *Automatic RP-18 chromatography*: For reverse-phase chromatography, an Intershim Puriflash 430 in combination with Chromabond Flash RS40 C_18_ec was used. *High-performance liquid chromatography*: HPLC was required for analytical studies and monitoring reactions. It was performed using a VWR-Hitachi LaChromElite HPLC system (L-2130, L-2200, L-2455) and EzChromElite software, equipped with a Nucleodur 100–5 C_18_ec or Nucleodur 100–5 C_8_ec (Macherey–Nagel). Acetonitrile for HPLC was obtained from VWR (HPLC grade) and ultrapure water using Sartorius Aurium pro (Sartopore 0.2 μm, UV detector). Tetra-*n*-butylammonium acetate solution (2 mM; TBAA, pH 6.3) or 10 mM triethylammonium acetate (TEAA, pH 6.2) were used for buffering. *Method A*: Nucleodur 100–5 C_18_ec; 0–20 min: TBAA buffer/acetonitrile gradient (5–80%); 20–30 min: buffer/acetonitrile (80%); 30–33 min: buffer/acetonitrile (80–5%); 33–38 min: buffer/acetonitrile (5%); flow: 1 ml min^−1^. *Method B*: Nucleodur 100–5 C_18_ec; 0–20 min: TEAA buffer/acetonitrile gradient (5–90%); 20–30 min: buffer/acetonitrile (90%); 30–33 min: buffer/acetonitrile (90–5%); 33–38 min: buffer/acetonitrile (5%); flow: 1 ml min^−1^. *Method C*: Nucleodur 100–5 C_8_ec; 0–25 min: TBAA buffer/acetonitrile gradient (5–80%); 25–30 min: buffer/acetonitrile (80%); 30–33 min: buffer/acetonitrile (80–5%); 33–38 min: buffer/acetonitrile (5%); flow: 1 ml min^−1^.

*Nuclear Magnetic Resonance*: NMR spectra were recorded at room temperature in an automation mode with a Varian Gemini 2000BB, Bruker Fourier 300, Bruker AMX 400, Bruker DRX 500 or Bruker AVIII 600. All ^1^H- and ^13^C-NMR chemical shifts (*δ*) are quoted in parts per million (p.p.m.) downfield from TMS and calibrated on solvent signal. The ^31^P-NMR chemical shifts (proton decoupled) are also quoted in p.p.m. using phosphoric acid as the external standard. *Mass spectrometry*: high resolution mass spectrometry (HRMS and electrospray ionization (ESI) mass spectra were acquired with a VG Analytical Finnigan ThermoQuest MAT 95 XL spectrometer. MALDI measurements (matrix: 9-aminoacridine (9AA)) were performed with a Bruker UltrafleXtreme spectrometer. *Infrared spectroscopy*: IR spectra were recorded on a Bruker Alpha P FT-IR at room temperature in the range of 400–4,000 cm^−1^.

### General procedure A: preparation of 4-acyloxybenzyl alcohols 9

4-Hydroxybenzyl alcohol **10** (1.1 eq.) and triethylamine (1.0 eq.) in THF were cooled down to 0 °C. The corresponding acyl chloride (1.0 eq.) in THF was added dropwise and the mixture stirred for 1–2 h. The precipitate was removed by filtration and the solvent evaporated in vacuum. The residue was diluted with CH_2_Cl_2_ and washed once with saturated sodium bicarbonate solution and once with water. The organic layer was dried with Na_2_SO_4_ and the solvent was removed in vaccum. The crude material was purified using column chromatography to give compound **9**.

The syntheses and characterization of 4-(hydroxymethyl)phenylalkanoates **9a**-**k** were described previously^33^.

### 4-(Hydroxymethyl)phenylmethylcarbonate **9l**

General procedure A with 4.0 g 4-hydroxybenzyl alcohol **10** (33 mmol, 1.1 eq.), 4.1 ml triethylamine (3.0 g, 30 mmol, 1.0 eq.) dissolved in 35 ml THF and dropwise addition of 2.3 ml methyl chloroformate (2.8 g, 30 mmol, 1.0 eq.) in 20 ml THF at 0 °C. Reaction time was 1.5 h at room temperature (rt). Column chromatography (petroleum ether 50–70/ethyl acetate 4:3 v/v). Yield: 4.6 g (25 mmol, 85%) colourless oil. TLC (petroleum ether 50–70/ethyl acetate 3:2 v/v): *R*_f_=0.35; ^1^H-NMR (400 MHz, dimethylsulphoxide (DMSO)-*d*_6_): *δ* 7.39–7.31 (*m*, 2H), 7.20–7.13 (*m*, 2H), 5.22 (*t*, *J*=5.7 Hz, 1H), 4.50 (*d*, *J*=5.8 Hz, 2H), 3.82 (*s*, 3H); ^13^C-NMR (101 MHz, DMSO-*d*_6_): *δ* 153.7, 149.5, 140.5, 127.5, 121.4, 62.3, 55.4; infrared red (IR): 3,375, 2,959, 2,873, 1,758, 1,254, 1,210 cm^−1^; HRMS (ESI^+^, *m/z*): [M+Na]^+^ calcd. for C_9_H_10_O_4_, 205.0471; found, 205.0337.

### 4-(Hydroxymethyl)phenyloctylcarbonate **9m**

General procedure A with 3.1 g 4-hydroxybenzyl alcohol **10** (25 mmol, 1.1 eq.), 3.1 ml triethylamine (2.3 g, 23 mmol, 1.0 eq.) dissolved in 35 ml THF and dropwise addition of 4.4 ml octyl chloroformate (4.4 g, 23 mmol, 1.0 eq.) in 20 ml THF at 0 °C. Reaction time was 1.5 h at rt. Column chromatography (petroleum ether 50–70/ethyl acetate 4:3 v/v). Yield: 5.3 g (19 mmol, 84%) colourless oil. TLC (PE/EE 3:1 v/v): *R*_f_=0.45; ^1^H-NMR (300 MHz, DMSO-*d*_6_): *δ* 7.39–7.31 (*m*, 2H), 7.21–7.12 (*m*, 2H), 5.22 (*t*, *J*=5.7 Hz, 1H), 4.49 (*d*, *J*=5.7 Hz, 2H), 4.18 (*t*, ^3^*J*_HH_=6.6 Hz, 2H), 1.72–1.58 (*m*, 2H), 1.41–1.18 (*m*, 10H), 0.87 (*t*, *J*=6.7 Hz, 3H); ^13^C-NMR (75 MHz, DMSO-*d*_6_): *δ* 153.2, 149.5, 140.4, 127.5, 120.8, 68.5, 62.3, 31.2, 28.6, 28.0, 25.2, 22.1, 28.6, 14.0; IR: 3,377, 2,955, 2,856, 1,758, 1,247, 1,210 cm^−1^; HRMS (ESI^+^, *m/z*): [M+Na]^+^ calcd. for C_16_H_24_O_4_, 303.1567; found, 303.1568.

### 4-(Hydroxymethyl)phenyldodecylcarbonate **9n**

General procedure A with 4.1 g 4-hydroxybenzyl alcohol **10** (33 mmol, 1.1 eq.), 4.0 ml triethylamine (2.9 g, 30 mmol, 1.0 eq.) dissolved in 40 ml THF and dropwise addition of 8.1 ml dodecyl chloroformate (7.5 g, 30 mmol, 1.0 eq.) in 20 ml THF at 0 °C. Reaction time was 1.5 h at rt. Column chromatography (petroleum ether (PE) 50–70/ethyl acetate (EE) 4:1 v/v). Yield: 7.7 g (23 mmol, 76%) colourless oil. TLC (PE/EE 3:1 v/v): *R*_f_=0.48; ^1^H-NMR (300 MHz, CDCl_3_): *δ* 7.42–7.33 (*m*, 2H), 7.21–7.12 (*m*, 2H), 4.68 (*s*, 2H), 4.24 (*t*, *J*=6.7 Hz, 2H), 1.81–1.69 (*m*, 2H), 1.47–1.19 (*m*, 18H), 0.88 (*t*, *J*=6.7 Hz, 3H); ^13^C-NMR (75 MHz, CDCl_3_): *δ* 153.9, 150.7, 138.8, 128.2, 121.1, 69.2, 64.8, 32.1, 29.8, 29.7, 29.6, 29.5, 29.3, 28.7, 25.8, 22.8, 28.6, 14.3; IR: 3,355, 2,917, 2,848, 1,747, 1,273 cm^−1^; HRMS (ESI^+^, *m/z*): [M+Na]^+^ calcd. for C_20_H_34_O_4_, 359.2193; found, 359.2195.

### 4-(((*tert*-Butyldimethylsilyl)oxy)methyl)phenol 11

4-Hydroxybenzyl alcohol **10** (5.1 g; 41 mmol, 1.0 eq.), dissolved in 40 ml DMF, was converted with 6.8 g *tert*-butyldimethylsilyl chloride (45 mmol, 1.1 eq.) and 6.1 g imidazole (89 mmol, 2.2 eq.). After 17 h the solvent was removed by evaporation. The crude product was dissolved in CH_2_Cl_2_ and washed with 0.1 M HCl. The organic phase was dried over Na_2_SO_4_, filtered and the solvent was removed by evaporation. Column chromatography (petroleum ether 50–70/ethyl acetate 6:1 v/v). Yield: 7.9 g (33 mmol, 81%) colourless oil. TLC (PE/EE 4:1 v/v): *R*_f_=0.34; ^1^H-NMR (300 MHz, DMSO-*d*_6_): *δ* 9.28 (*s*, 1H), 7.12–7.06 (*m*, 2H), 6.74–6.68 (*m*, 2H), 4.56 (*s*, 2H), 0.87 (*s*, 9H), 0.04 (*s*, 6H); ^13^C-NMR (75 MHz, DMSO-*d*_6_): *δ* 139.2, 131.6, 127.5, 114.6, 64.0, 25.6, 17.6, −5.4; IR: 3,355, 2,954, 2,857, 1,707, 1,515 cm^−1^; HRMS (ESI^+^, *m/z*): [M+Na]^+^ calcd. for C_13_H_22_O_2_Si, 261.1287; found, 261.1285.

### 4-(((*tert*-Butyldimethylsilyl)oxy)methyl)phenyl-(4-nitrophenyl)-carbonate 12

Compound **11** (7.6 g; 32 mmol, 1.0 eq.) was dissolved in 100 ml CH_2_Cl_2_. 4-Nitrophenyl chloroformate (12.8 g; 64 mmol, 2.0 eq.) was added slowly to the reaction flask and the mixture was kept for 1 h at rt. Then, for consumption of the excess of chloroformate and for facilitation of purification, 7.5 ml tri(ethylene glycol) monomethyl ether (7.9 g, 48 mmol, 1.5 eq.) was added. After 20 min the solution was diluted with CH_2_Cl_2_ and washed with 1 M HCl. The organic phase was dried over Na_2_SO_4_, filtered and the solvent was removed by evaporation. Column chromatography (petroleum ether 50–70/ethyl acetate 6:1 v/v). Yield: 6.8 g (17 mmol, 53%) colourless solid. TLC (PE/EE 6:1 v/v): *R*_f_=0.73; ^1^H-NMR (400 MHz, DMSO-*d*_6_): *δ* 8.38–8.33 (*m*, 2H), 7.73–7.67 (*m*, 2H), 7.43–7.36 (*m*, 4H), 4.73 (*s*, 2H), 0.91 (*s*, 9H), 0.09 (*s*, 6H); ^13^C-NMR (101 MHz, DMSO-*d*_6_): *δ* 155.2, 150.7, 149.3, 145.4, 139.6, 127.2, 125.4, 122.7, 120.9, 63.6, 25.8, 18.0, −5.3; IR: 2,954, 2,929, 2,857, 1,768, 1,265 cm^−1^; HRMS (ESI^+^, *m/z*): [M+Na]^+^ calcd. for C_20_H_25_NO_6_Si, 426.1349; found, 426.1340.

### *tert*-Butylmethyl(2-(methylamino)ethyl)-carbamate 13

A flask containing 4.6 ml *N,Ń*-dimethylethylendiamine (3.7 g, 42 mmol, 3.8 eq.) in 40 ml CH_2_Cl_2_ was cooled to 0 °C. A solution of 2.4 g di-*tert*-butyl dicarbonate (11 mmol, 1.0 eq.) in 20 ml CH_2_Cl_2_ was added dropwise and, following the reaction mixture, was allowed to warm to rt. After 16 h, the solvent was removed in vacuum. The product was extracted with ethyl acetate/water (2:1 v/v) and washed with saturated aqueous NaHCO_3_. The organic phase was dried over Na_2_SO_4_, filtered and the solvent was removed by evaporation. Yield: 1.6 g (8.6 mmol, 78%) yellowish oil. ^1^H-NMR (600 MHz, CDCl_3_): *δ* 3.35–3.25 (*m*, 2H, rotamers), 2.85 (s, 3H), 2.70 (t, *J*=6.7 Hz, 2H), 2.42 (s, 3H), 1.43 (s, 9H); ^13^C-NMR (151 MHz, CDCl_3_): *δ* 156.1, 79.5, 49.8, 48.8+48.3 (rotamers), 36.4, 34.9+34.8 (rotamers), 28.6; IR: 2,974, 2,931, 1,687, 1,154 cm^−1^; HRMS (ESI^+^, *m/z*): [M+Na]^+^ calcd. for C_9_H_20_N_2_O_2_, 211.1417; found, 211.1411.

### 4-(Hydroxymethyl)phenyl-methyl(2-(methylamino)ethyl)-carbamate 14

At 0 °C to a stirred solution of 1.6 g compound **13** (8.6 mmol, 1.2 eq.), 1.9 ml di*iso*propylethylamine (1.4 g, 11 mmol, 1.6 eq.) and catalytic amounts of 4-dimethylaminophenol in 30 ml toluene 2.8 g activated carbonate **12** (7.0 mmol, 1.0 eq.) was added. The reaction mixture was allowed to warm to rt and stirred for 16 h. The solution was diluted with ethyl acetate and washed with 1 M HCl, followed by saturated aqueous NaHCO_3_. The organic phase was dried over Na_2_SO_4_, filtered and the solvent was removed by evaporation. For deprotection the residue was redissolved in a mixture of CH_2_Cl_2_/trifluoroacetic acid (1:1 v/v). After 1 h the volatile components were removed by evaporation and the residue was co-evaporated twice with toluene. The crude material was purified by automatic RP-18 chromatography (water/acetontrile gradient). Yield: 1.6 g (6.9 mmol, 98%) yellowish oil. TLC (CH_2_Cl_2_/MeOH 4:1 v/v): *R*_f_=0.67; ^1^H-NMR (400 MHz, DMSO-*d*_6_): *δ* 8.72, 8.62 (br.*s*, 2H, rotamers), 7.34–7.28 (*m*, 2H), 7.14–7.07 (*m*, 2H), 4.48 (*s*, 2H), 3.69, 3.57 (*t*, *J*=6.0 Hz, 2H, rotamers), 3.24–3.10 (*m*, 2H, rotamers), 3.05, 2.93 (*s*, 3H, rotamers), 2.68–2.57 (*m*, 3H, rotamers); ^13^C-NMR (101 MHz, DMSO-*d*_6_): *δ* 154.7+153.8 (rotamers), 149.9, 139.7, 127.2, 121.7+121.4 (rotamers), 62.4, 46.3+46.0 (rotamers), 45.2, 34.6+34.4 (rotamers), 32.9+32.8 (rotamers); IR: 3,401, 2,975, 2,871, 1,671, 1,172 cm^−1^; HRMS (ESI^+^, *m/z*): [M+Na]^+^ calcd. for C_12_H_18_N_2_O_3_, 239.1390; found, 239.1391.

### General procedure B: preparation of bis-methylcarbamates **15**

4-(Hydroxymethyl)phenylcarbamate **14** (1.0 eq.) was dissolved in THF and cooled down to 0 °C. After addition of imidazole (1.3 eq.) and TMSCl (1.2 eq.) the mixture was stirred for 2 h. Triethylamine (3.1 eq.) and the corresponding alkyl chloroformate (3.0 eq.) were added. The reaction was kept for 1.5 h at rt and then diluted with 30 ml of 1 vol% concentrated (12 M) HCl in EtOH for desilylation. The solution was stirred for 1 h and the solvent evaported in vacuum. The residue was dissolved in CH_2_Cl_2_ and washed with saturated aqueous NaHCO_3_. The organic phase was dried over Na_2_SO_4_, filtered and the solvent was removed in vacuum. The crude material was purified using column chromatography.

### *n*-Butyl-(4-(hydroxymethyl)phenyl)ethane-1,2-diylbis(methylcarbamate) **15o**

General procedure B with 2.1 g **14** (8.8 mmol, 1.0 eq.), 0.78 g imidazole (11 mmol, 1.3 eq.), 1.3 ml TMSCl (1.1 g, 11 mmol, 1.2 eq.), 3.8 ml triethylamine (2.8 g, 27 mmol, 3.1 eq.), 3.4 ml butyl chloroformate (3.6 g, 26 mmol, 3.0 eq.) dissolved in 15 ml THF. Column chromatography (petroleum ether 50–70/ethyl acetate 1:1 v/v). Yield: 1.8 g (19 mmol, 59%) colourless oil. TLC (CH_2_Cl_2_/MeOH 9:1 v/v): *R*_f_=0.56; ^1^H-NMR (400 MHz, DMSO-*d*_6_): *δ* 7.34–7.27 (*m*, 2H), 7.04–6.97 (*m*, 2H), 5.17 (*t*, *J*=5.6 Hz, 1H), 4.48 (*d*, *J*=4.3 Hz, 2H), 4.02–3.91 (*m*, 2H), 3.57–3.40 (*m*, 4H, rotamers), 3.02, 2.90 (*s*, 3H, rotamers), 2.90–2.82 (*m*, 3H, rotamers), 1.60–1.44 (*m*, 2H, rotamers), 1.39–1.22 (*m*, 2H, rotamers), 0.87, 0.80 (*t*, *J*=7.3 Hz, 3H, rotamers); ^13^C-NMR (101 MHz, DMSO-*d*_6_): *δ* 155.8, 153.9, 150.0, 139.3, 127.2, 121.4+121.3 (rotamers), 64.5+64.4 (rotamers), 62.4, 46.5+46.2 (rotamers), 46.0+45.6 (rotamers), 35.2+35.0 (rotamers), 34.7+34.5 (rotamers), 30.6+30.6 (rotamers), 18.6, 13.6; IR: 3,445, 2,958, 2,933, 1,694, 1,202 cm^−1^; HRMS (ESI^+^, *m/z*): [M+H]^+^ calcd. for C_17_H_26_N_2_O_5_, 339.1914; found, 339.1918.

### *n*-Octyl-(4-(hydroxymethyl)phenyl)ethane-1,2-diylbis(methylcarbamate) **15p**

General procedure B with 0.86 g **14** (3.6 mmol, 1.0 eq.), 0.32 g imidazole (4.7 mmol, 1.3 eq.), 0.55 ml TMSCl (0.47 g, 4.3 mmol, 1.2 eq.), 1.6 ml triethylamine (1.1 g, 11 mmol, 3.1 eq.), 2.1 ml octyl chloroformate (2.1 g, 11 mmol, 3.0 eq.) dissolved in 5 ml THF. Column chromatography (petroleum ether 50–70/ethyl acetate 1:1 v/v). Yield: 0.72 g (1.8 mmol, 51%) colourless oil. TLC (CH_2_Cl_2_/MeOH 9:1 v/v): *R*_f_=0.60; ^1^H-NMR (400 MHz, DMSO-*d*_6_): *δ* 7.34–7.27 (*m*, 2H), 7.02–6.98 (*m*, 2H), 5.17 (*t*, *J*=5.7 Hz, 1H), 4.47 (*d*, *J*=5.6 Hz, 2H), 4.01–3.90 (*m*, 2H), 3.57–3.39 (*m*, 4H, rotamers), 3.02, 2.90 (*s*, 3H, rotamers), 2.90–2.81 (*m*, 3H, rotamers), 1.62–1.44 (*m*, 2H, rotamers), 1.36–1.12 (*m*, 10H), 0.89–0.80 (*m*, 3H, rotamers); ^13^C-NMR (101 MHz, DMSO-*d*_6_): *δ* 155.7, 154.0, 150.0, 139.2, 127.2, 121.4+121.3 (rotamers), 64.8+64.7 (rotamers), 62.4, 46.5+46.2 (rotamers), 45.9+45.5 (rotamers), 34.6+34.5 (rotamers), 31.2, 28.7, 28.6+28.6 (rotamers), 25.4, 22.0, 13.9; IR: 3,447, 2,926, 2,856, 1,698, 1,203 cm^−1^; HRMS (ESI^+^, *m/z*): [M+H]^+^ calcd. for C_21_H_34_N_2_O_5_, 395.2540; found, 395.2540.

### *n*-Dodecyl-(4-(hydroxymethyl)phenyl)ethane-1,2-diylbis(methylcarbamate) **15q**

General procedure B with 0.95 g **14** (4.0 mmol, 1.0 eq.), 0.35 g imidazole (5.2 mmol, 1.3 eq.), 0.61 ml TMSCl (0.52 g, 4.8 mmol, 1.2 eq.), 1.7 ml triethylamin (1.3 g, 12 mmol, 3.1 eq.), 3.2 ml dodecyl chloroformate (3.0 g, 12 mmol, 3.0 eq.) dissolved in 15 ml THF. Column chromatography (petroleum ether 50–70/ethyl acetate 1:1 v/v). Yield: 0.75 g (1.7 mmol, 42%) colourless oil. TLC (CH_2_Cl_2_/MeOH 9:1 v/v): *R*_f_=0.65; ^1^H-NMR (400 MHz, CDCl_3_): *δ* (p.p.m.)=7.36–7.31 (*m*, 2H), 7.10–7.05 (*m*, 2H), 4.64 (s, 2H), 4.12–4.02 (*m*, 2H), 3.63–3.44 (*m*, 4H, rotamers), 3.15–2.89 (s, 6H, rotamers), 1.68–1.54 (*m*, 2H, rotamers), 1.43–1.13 (*m*, 10H), 0.88 (*t*, *J*=6.8 Hz, 3H); ^13^C-NMR (101 MHz, CDCl_3_): *δ* 157.1, 154.9, 151.0, 138.3, 128.0, 121.9+121.3 (rotamers), 65.9+65.8 (rotamers), 64.9, 47.4+47.3 (rotamers), 47.1+46.4 (rotamers), 35.4+35.3 (rotamers), 32.1, 29.8, 29.7, 29.7, 29.5+29.5 (rotamers), 29.4, 29.2, 29.2, 26.1, 22.8, 14.2; IR: 3,459, 2,923, 1,699, 1,203 cm^−1^; HRMS (ESI^+^, *m/z*): [M+H]^+^ calcd. for C_25_H_42_N_2_O_5_, 451.3172; found, 451.3167.

### General procedure C: preparation of bis-(4-acyloxybenzyl)-*N,N*-di*iso*propylphosphoramidites 5

Dichloro-*N,N*-di*iso*propylphosphoramidite (1.0 eq.) was dissolved in THF and cooled to 0 °C. Triethylamine (2.3 eq.) and the corresponding 4-acyloxybenzylalcohol **9** (2.1–2.2 eq.) in THF were added dropwise. The reaction mixture was kept at 0 °C for 18–24 h. After filtration, the solvent was removed by evaporation. The crude products were purified either using column chromatography or using preparative TLC (chromatotron).

The syntheses and characterization of Bis-(4-acyloxybenzyl)-*N,N*-di*iso*propylphosphoramidite **5a–k** were described previously^33^.

### Bis-(4-methyloxycarbonyloxybenzyl)-*N*,*N*-di*iso*propylaminophosphoramidite **5l**

General procedure C with 0.74 g dichloro-*N,N*-di*iso*propylphosphoramidite (3.7 mmol, 1.0 eq.) dissolved in 15 ml THF, 1.2 ml triethylamine (0.85 g, 8.4 mmol, 2.3 eq.) and 1.5 g 4-(hydroxymethyl)phenylmethylcarbonate (8.2 mmol, 2.2 eq.) in 15 ml THF. The crude product was purified by preparative TLC (petroleum ether 50–70/ethyl acetate 4:1 v/v+5% Et_3_N). Yield: 1.5 g (3.0 mmol, 82%) colourless oil. TLC (PE/EE 1:1 v/v+5% Et_3_N): *R*_f_=0.77; ^1^H-NMR (300 MHz, DMSO-*d*_6_): *δ* 7.42–7.34 (*m*, 4H), 7.18–7.07 (*m*, 4H), 4.78–4.61 (*m*, 4H), 3.82 (*s*, 6H), 3.72–3.57 (*m*, 2H), 1.16 (*d*, *J*=6.8 Hz, 12H); ^13^C-NMR (75 MHz, DMSO-*d*_6_): *δ* 153.7, 149.9, 137.2 (*d*, *J*=7.4 Hz), 128.0, 121.1, 64.1 (*d*, *J*=18.1 Hz), 55.4, 42.6 (*d*, *J*=12.5 Hz), 24.4 (*d*, *J*=6.7 Hz); ^31^P-NMR (202 MHz, DMSO-*d*_6_): *δ* 147.5; IR: 2,965, 2,916, 1,760, 1,200, 1,126 cm^−1^; HRMS (ESI^+^, *m/z*): [M+H]^+^ calcd. for C_24_H_32_NO_8_P, 494.1938; found, 494.2276.

### Bis-(4-octyloxycarbonyloxybenzyl)-*N*,*N*-di*iso*propylaminophosphoramidite **5m**

General procedure C with 0.76 g dichloro-*N,N*-di*iso*propylphosphoramidite (3.8 mmol, 1.0 eq.) dissolved in 15 ml THF, 1.3 ml triethylamine (0.88 g, 8.7 mmol, 2.3 eq.) and 2.3 g 4-(hydroxymethyl)phenyloctylcarbonate (8.3 mmol, 2.2 eq.) in 15 ml THF. The crude product was purified by preparative TLC (petroleum ether 50–70/ethyl acetate 6:1 v/v+5% Et_3_N). Yield: 2.2 g (3.2 mmol, 83%) colourless oil. TLC (PE/EE 6:1 v/v+5% Et_3_N): *R*_f_=0.43; ^1^H-NMR (300 MHz, CDCl_3_): *δ* 7.39–7.31 (*m*, 4H), 7.20–7.13 (*m*, 4H), 4.80–4.62 (*m*, 4H), 4.24 (*t*, *J*=6.7 Hz, 4H), 3.69 (*d*quint, *J*=10.0 Hz, *J*=6.8 Hz, 2H), 1.82–1.66 (*m*, 4H), 1.48–1.23 (*m*, 16H), 1.20 (*d*, *J*=6.8 Hz, 12H), 0.89 (*t*, *J*=6.7 Hz, 6H); ^13^C-NMR (75 MHz, CDCl_3_): *δ* 153.9, 150.4, 137.4 (*d*, *J*=7.5 Hz), 128.2, 121.0, 69.1, 64.9 (*d*, *J*=18.7 Hz), 42.2 (*d*, *J*=12.6 Hz), 31.9, 29.3, 29.3, 25.8, 22.8, 28.7, 24.8 (*d*, *J*=7.6 Hz), 14.2; ^31^P-NMR (202 MHz, CDCl_3_): *δ* 148.0; IR: 2,961, 2,926, 1,759, 1,215 cm^−1^; HRMS (ESI^+^, *m/z*): [M+H]^+^ calcd. for C_38_H_60_NO_8_P, 690.4129; found, 690.4086.

### Bis-(4-dodecyloxycarbonyloxybenzyl)-*N*,*N*-di*iso*propylaminophosphoramidite **5n**

General procedure C with 0.50 g dichloro-*N,N*-di*iso*propylphosphoramidite (2.5 mmol, 1.0 eq.) dissolved in 14 ml THF, 0.80 ml triethylamine (0.58 g, 5.8 mmol, 2.3 eq.) and 1.8 g 4-(hydroxymethyl)phenyldodecylcarbonate (5.2 mmol, 2.1 eq.) in 10 ml THF. The crude product was purified using preparative TLC (petroleum ether 50–70/ethyl acetate 10:1 v/v+5% Et_3_N). Yield: 1.8 g (2.2 mmol, 88%) colourless solid. TLC (PE/EE 6:1 v/v+5% Et_3_N): *R*_f_=0.53; ^1^H-NMR (300 MHz, CDCl_3_): *δ* 7.39–7.32 (*m*, 4H), 7.17–7.09 (*m*, 4H), 4.80–4.62 (*m*, 4H), 4.24 (*t*, *J*=6.7 Hz, 4H), 3.75–3.57 (*m*, 2H), 1.80–1.67 (*m*, 4H), 1.47–1.23 (*m*, 36H), 1.20 (*d*, *J*=6.8 Hz, 12H), 0.88 (*t*, *J*=6.7 Hz, 6H); ^13^C-NMR (75 MHz, CDCl_3_): *δ* 153.9, 150.4, 137.4 (*d*, *J*=7.8 Hz), 128.2, 121.0, 69.1, 64.9 (*d*, *J*=18.2 Hz), 43.2 (*d*, *J*=12.5 Hz), 32.1, 29.8, 29.7, 29.6, 29.5, 29.4, 25.8, 22.8, 28.6, 24.8 (*d*, *J*=7.4 Hz), 14.3; ^31^P-NMR (162 MHz, CDCl_3_): *δ* 148.0; IR: 2,961, 2,923, 2,853, 1,760, 1,216 cm^−1^; HRMS (ESI^+^, *m/z*): [M+H]^+^ calcd. for C_46_H_76_NO_8_P, 802.5381; found, 802.5352.

### Bis-(4-(butyl-ethan-1,2-diylbis(methylcarbamate))-oxybenzyl)-*N*,*N*-di*iso*propylaminophosphoramidite **5o**

General procedure C with 0.20 g dichloro-*N,N*-di*iso*propylphosphoramidite (0.99 mmol, 1.0 eq.) dissolved in 4 ml THF, 0.32 ml triethylamine (0.23 g, 2.3 mmol, 2.3 eq.) and 0.75 g compound **15o** (2.2 mmol, 2.2 eq.) in 5 ml THF. The crude product was purified using column chromatography (petroleum ether 50–70/ethyl acetate 1:1 v/v+5% Et_3_N). Yield: 0.49 g (0.61 mmol, 62%) colourless oil. TLC (PE/EE 1:1 v/v+5% Et_3_N): *R*_f_=0.30; ^1^H-NMR (400 MHz, CDCl_3_): *δ* 7.39–7.30 (*m*, 4H), 7.10–7.01 (*m*, 4H), 4.78–4.60 (*m*, 4H), 4.14–4.02 (*m*, 4H), 3.76–3.61 (*m*, 2H), 3.62–3.42 (*m*, 8H, rotamers), 3.15–2.89 (*m*, 12H, rotamers), 1.65–1.56 (*m*, 4H, rotamers), 1.46–1.30 (*m*, 4H, rotamers), 1.19 (*d*, *J*=6.9 Hz, 12H), 0.93, 0.88 (*t*, *J*=7.4 Hz, 6H, rotamers); ^13^C-NMR (101 MHz, CDCl_3_): *δ* 156.9, 154.8, 150.5, 136.5, 128.0, 121.6+121.6 (rotamers), 65.7+65.6 (rotamers), 65.0 (*d*, *J*=18.1 Hz), 47.8+47.4 (rotamers), 47.0+46.4 (rotamers), 43.3 (*d*, *J*=12.2 Hz), 35.4+35.3 (rotamers), 35.2+34.8 (rotamers), 31.3+31.3 (rotamers), 24.8 (*d*, *J*=7.5 Hz), 19.3, 13.9; ^31^P-NMR (162 MHz, CDCl_3_): *δ* 147.4; IR: 2,962, 2,932, 2,871, 1,719, 1,695, 1,199 cm^−1^; HRMS (ESI^+^, *m/z*): [M+H]^+^ calcd. for C_40_H_64_N_5_O_10_P, 806.4464; found, 806.4440.

### Bis-(4-(octyl-ethan-1,2-diylbis(methylcarbamate))-oxybenzyl)-*N*,*N*-di*iso*propylaminophosphoramidite **5p**

General procedure C with 0.14 g dichloro-*N,N*-di*iso*propylphosphoramidite (0.71 mmol, 1.0 eq.) dissolved in 10 ml THF, 0.23 ml triethylamine (0.17 g, 1.6 mmol, 2.3 eq.) and 0.59 g compound **15p** (1.5 mmol, 2.1 eq.) in 5 ml THF. The crude product was purified using column chromatography (petroleum ether 50–70/ethyl acetate 1:1 v/v+5% Et_3_N). Yield: 0.59 g (0.64 mmol, 90%) colourless oil. TLC (PE/EE 1:1 v/v+5% Et_3_N): *R*_f_=0.41; ^1^H-NMR (400 MHz, CDCl_3_): *δ* 7.37–7.29 (*m*, 4H), 7.10–7.02 (*m*, 4H), 4.78–4.61 (*m*, 4H), 4.12–4.02 (*m*, 4H), 3.74–3.63 (*m*, 2H), 3.62–3.43 (*m*, 8H, rotamers), 3.15–2.89 (*m*, 12H, rotamers), 1.69–1.55 (*m*, 4H, rotamers), 1.40–1.19 (*m*, 36H, rotamers), 1.19 (*d*, *J*=6.8 Hz, 12H), 0.87 (*t*, *J*=6.8 Hz, 6H, H-n, rotamers); ^13^C-NMR (101 MHz, CDCl_3_): *δ* 156.6, 154.8, 150.7, 136.6, 128.0, 121.6+121.5 (rotamers), 65.9+65.8 (rotamers), 65.0 (*d*, *J*=18.0 Hz), 47.6+47.4 (rotamers), 47.0+46.4 (rotamers), 43.2 (*d*, *J*=12.6 Hz), 35.4+35.3 (rotamers), 35.1+34.8 (rotamers), 31.9, 29.4, 29.3, 29.2+29.2 (rotamers), 26.1, 24.8 (*d*, *J*=7.2 Hz), 22.8, 14.2; ^31^P-NMR (162 MHz, CDCl_3_): *δ* 147.7; IR: 2,958, 2,927, 2,856, 1,722, 1,699, 1,202 cm^−1^; HRMS (ESI^+^, *m/z*): [M+H]^+^ calcd. for C_48_H_80_N_5_O_10_P, 918.5716; found, 918.6124.

### Bis-(4-(dodecyl-ethan-1,2-diylbis(methylcarbamate))-oxybenzyl)-*N*,*N*-di*iso*propylaminophosphoramidite **5q**

General procedure C with 0.15 g dichloro-*N,N*-di*iso*propylphosphoramidite (0.75 mmol, 1.0 eq.) dissolved in 3 ml THF, 0.24 ml triethylamine (0.17 g, 1.7 mmol, 2.3 eq.) and 0.71 g compound **15q** (1.6 mmol, 2.1 eq.) in 5 ml THF. The crude product was purified using column chromatography (petroleum ether 50–70/ethyl acetate 1:1 v/v+5% Et_3_N). Yield: 0.37 g (0.36 mmol, 48%) colourless oil. TLC (PE/EE 1:1 v/v+5% Et_3_N): *R*_f_=0.50; ^1^H-NMR (600 MHz, CDCl_3_): *δ* 7.36–7.30 (*m*, 4H), 7.08–7.03 (*m*, 4H), 4.76–4.62 (*m*, 4H), 4.11–4.02 (*m*, 4H), 3.73–3.62 (*m*, 2H), 3.61–3.42 (*m*, 8H, rotamers), 3.14–2.90 (*m*, 12H, rotamers), 1.67–1.56 (*m*, 4H, rotamers), 1.37–1.21 (*m*, 36H), 1.19 (*d*, *J*=6.8 Hz, 12H), 0.87 (*t*, *J*=6.9 Hz, 6H, rotamers); ^13^C-NMR (101 MHz, CDCl_3_): *δ* 157.0, 154.7, 150.7+150.6 (rotamers), 136.6, 128.0+128.0 (*d*, *J*=2.6 Hz), 121.6+121.5 (rotamers), 65.9+65.8 (rotamers), 65.0 (*d*, *J*=18.3 Hz), 47.4+47.3 (rotamers), 46.9+46.4 (rotamers), 43.2 (*d*, *J*=12.3 Hz), 35.4, 35.3 (rotamers), 35.0+34.6 (rotamers), 32.0, 29.8, 29.7, 29.7, 29.7, 29.5, 29.4, 29.2+29.2 (rotamers), 26.1, 24.8 (*d*, *J*=7.4 Hz), 22.8, 14.2; ^31^P-NMR (162 MHz, CDCl_3_): *δ* 147.3; IR: 2,923, 2,853, 1,721, 1,699, 1,200 cm^−1^; HRMS (ESI^+^, *m/z*): [M+H]^+^ calcd. for C_48_H_80_N_5_O_10_P, 1030.6982; found, 1030.6968.

### 5-Chloro-*cyclo*Sal-3′-deoxy-2′,3′-didehydrothymidine monophosphate 7

To a suspension of 0.50 g d4T **1** (2.2 mmol, 1.0 eq.) in 8 ml, acetonitrile was added 0.53 ml di*iso*propylethlyamine (0.54 g, 3.1 mmol, 1.4 eq.) followed by 0.60 g 5-chlorosaligenylchlorophosphite **8** (2.7 mmol, 1.2 eq.). The reaction mixture was stirred for 3 h and subsequently cooled to 0 °C. By addition of a 5.5-M solution of 0.57 ml *tert*-butylhydroperoxide in *n*-decane (3.1 mmol, 1.4 eq.) the phosphite was oxidized for 20 min. The solvent was removed in vacuum. The residue was redissolved in CH_2_Cl_2_ and washed with 1 M ammonium acetate solution. The organic phase was dried over Na_2_SO_4_, filtered and the solvent was removed by evaporation. The crude product was purified using column chromatography (CH_2_Cl_2_/MeOH 9:1 v/v). Yield: 0.91 g (2.1 mmol, 97%) colourless foam as a mixture of two diastereomers. TLC (CH_2_Cl_2_/MeOH 9:1 v/v): *R*_f_=0.29; ^1^H-NMR (500 MHz, CDCl_3_): *δ* 8.92, 8.92 (br.s, 1H, diasteromers), 7.33–7.26 (*m*, 1H, ds), 7.20, 7.18 (*s*, 1H, ds), 7.12–7.07 (*m*, 1H, ds), 7.04–6.95 (*m*, 2H, ds), 6.40–6.32 (*m*, 1H, ds), 5.94, 5.94 (*d*, *J*=5.8 Hz, ds), 5.43–5.18 (*m*, 2H, ds), 5.06–4.99 (*m*, 1H, ds), 4.47–4.30 (*m*, 2H, ds), 1.82, 1.73 (*s*, 3H, ds); ^13^C-NMR (126 MHz, CDCl_3_): *δ* 163.9+163.8 (diastereomers), 150.9+150.8 (ds), 148.7+148.7 (ds), 135.8+135.6 (ds), 132.8+132.7 (ds), 130.3+130.3 (ds), 130.1+130.1 (ds), 128.1+128.0 (ds), 125.7+125.6 (ds), 122.3+122.3 (ds), 120.1+120.0 (*d*, *J*=5.3 Hz, ds), 111.5+111.4 (ds), 90.0+89.9 (ds), 84.6+84.5 (ds), 68.7+68.6 (ds), 68.1+67.9 (*d*, *J*=6.7 Hz, ds), 12.4+12.3 (ds); ^31^P-NMR (162 MHz, CDCl_3_): *δ* −9.80, −9.87 (*s*, diastereomers); IR: 3,168, 3,050, 2,886, 1,684 cm^−1^; HRMS (ESI^+^, *m/z*): [M+H]^+^ calcd. for C_17_H_16_ClN_2_O_7_P, 449.0276; found, 449.0287.

### 3′-Deoxy-2′,3′-didehydrothymidinediphosphate **6** (d4TDP, tetra-*n*-butylammonium salt)

*cyclo*Sal-triester **7** (1.1 g; 2.5 mmol, 1.0 eq.) was dissolved in 10 ml DMF and added dropwise to a solution of 2.1 g mono-(tetra-*n*-butylammonium)-phosphate (6.2 mmol, 2.5 eq.) in 12 ml DMF. After 16 h, the solvent was removed in vacuum. The residue was extracted with ethyl acetate/water followed by freeze-drying of the aqueous phase. The crude product was purified using automatic RP-18 chromatography (water/acetonitrile gradient: 5–100%, 0–90 min, flow 1 ml min^−1^). The purification had to repeat for complete removement of the excess of the monophosphate salt. Yield: 0.78 g (0.90 mmol, 36%, 2 × Bu_4_N^+^) colourless solid. TLC (*iso*propanol/NH_3_/water 4:1:2.5 v/v/v): *R*_f_=0.16; ^1^H-NMR (300 MHz, D_2_O): *δ* 7.60 (*d*, *J*=1.3 Hz, 1H), 6.93 (dt, *J*=3.3 Hz, *J*=1.7 Hz, 1H), 6.48 (dt, *J*=6.2 Hz, *J*=1.8 Hz, 1H), 5.91 (ddd, *J*=6.1 Hz, *J*=2.4 Hz, *J*=1.4 Hz, 1H), 5.10–5.04 (*m*, 1H), 4.11 (dt, *J*=6.2 Hz, *J*=3.3 Hz, 2H), 3.25–3.05 (*m*, 16H), 1.86 (*d*, *J*=1.3 Hz, 3H), 1.73–1.49 (*m*, 16H), 1.22 (sext, *J*=7.4 Hz, 16H), 0.91 (*t*, *J*=7.3 Hz, 24H); ^13^C-NMR (75 MHz, D_2_O): *δ* 166.8, 152.2, 138.1, 134.2, 125.1, 111.2, 89.8, 85.8 (*d*, *J*=8.5 Hz), 66.2 (*d*, *J*=5.6 Hz), 58.0, 23.0, 19.0, 12.8, 11.4; ^31^P-NMR (162 MHz, D_2_O): *δ* −8.32 (*d*, *J*=21.7 Hz), −11.23 (*d*, *J*=21.7 Hz); IR: 3,220, 1,645, 1,486 cm^−1^; MALDI-MS (ESI^+^, *m/z*): [M-H]^−^ calcd. for C_10_H_14_N_2_O_10_P_2_, 383.005; found, 382.928.

### General procedure D: preparation of γ-bis(4-alkanoyloxybenzyl)-d4TTPs **3**

D4TDP **6** (1.0 eq.) were once co-evaporated with DMF and then dissolved in acetonitrile. Phosphoramidites **5** (1.7–2.0 eq.) were added and the solvent removed in vacuum quantitatively. The residue was redissolved in a minimum of acetonitrile or in a mixture of acetonitrile/THF (1:1), and the reaction was started by addition of a 0.25-M solution of DCI in acetonitrile (1.2–1.4 eq.). After stirring for 1 min the reaction was cooled to −10 °C, and a 5.5 M solution of *t*-BuOOH in *n*-decane (2.1–2.2 eq.) was added for oxidation. The mixture was stirred for 20 min and the volatile components were removed in vacuum. The reaction was monitored with HPLC. If the conversion of d4TDP was not complete, the procedure was repeated as described above. The crude products were purified by automatic RP-18 flash chromatography followed by an ion exchange to the ammonium form with Dowex 50WX8 cation exchange resin and a second RP-18 chromatography (**3a–g**,**l–q**). For elution water/acetonitrile (5–100%, 0–40 min, flow 1 ml min^−1^) or water/THF gradients (5–80%, 0–40 min, flow 1 ml min^−1^ were used. Product-containing fractions were pooled and the organic solvent evaporated. The remaining aqueous solutions were freeze-dried and the desired product obtained as colourless solids.

### γ-Bis-(4-acetyloxybenzyl)-d4TTP **3a** (ammonium salt)

General procedure D with 86 mg d4TDP **6** (99 μmol, 1.0 eq.), 92 mg **5a** (0.20 mmol, 2.0 eq.), 0.55 ml 0.25 M solution of DCI in acetonitrile (0.14 mmol, 1.4 eq.), 40 μl 5.5 M solution of *t*-BuOOH in *n*-decane (0.22 mmol, 2.2 eq.) in 0.7 ml acetonitrile. The crude product was purified using automatic RP-18 chromatography (water/acetonitrile gradient). Yield: 46 mg (58 μmol, 59%) colourless solid. UV (HPLC): *λ*_max_=265 nm; HPLC: *t*_R_=11.00 min (method A); 8.75 min (method B); ^1^H-NMR (400 MHz, CD_3_OD): *δ* 7.67 (*d*, *J*=1.3 Hz, 1H), 7.46–7.40 (*m*, 4H), 7.13–7.06 (*m*, 4H), 6.94 (dt, *J*=3.4 Hz, *J*=1.8 Hz, 1H), 6.48 (dt, *J*=5.9 Hz, *J*=1.9 Hz, 1H), 5.82 (dt, *J*=5.9 Hz, *J*=1.9 Hz, 1H), 5.18 (*d*, *J*=8.1 Hz, 4H), 4.99–4.93 (*m*, 1H), 4.32–4.17 (*m*, 2H), 2.30 (*s*, 6H), 1.92 (*d*, *J*=1.3 Hz, 3H); ^13^C-NMR (101 MHz, CD_3_OD): *δ* 171.1, 166.5, 152.8, 152.3, 138.7, 134.9, 134.9 (d, *J*=6.8 Hz), 130.4 (*d*, *J*=2.7 Hz), 127.3, 122.9, 112.0, 90.8, 87.1 (*d*, *J*=8.8 Hz), 70.3 (*d*, *J*=6.1 Hz), 67.6, 20.9, 12.5; ^31^P-NMR (162 MHz, CD_3_OD): *δ* −11.69 (*d*, *J*=19.4 Hz), −13.11 (*d*, *J*=17.1 Hz), −23.51 (*t*, *J*=17.8 Hz); IR: 3,191, 2,988, 1,756, 1,687, 1,193 cm^−1^; MALDI-MS (*m/z*): [M-H]^−^ calcd. for C_28_H_31_N_2_O_17_P_3_, 759.076; found, 759.131.

### γ-Bis-(4-propanoyloxybenzyl)-d4TTP 3b (ammonium salt)

General procedure D with 87 mg d4TDP **6** (0.10 mmol, 1.0 eq.), 93 mg **5b** (0.20 mmol, 2.0 eq.), 0.48 ml 0.25 M solution of DCI in acetonitrile (0.12 mmol, 1.2 eq.), 38 μl 5.5 M solution of *t*-BuOOH in *n*-decane (0.21 mmol, 2.1 eq.) in 0.5 ml acetonitrile. The crude product was purified using automatic RP-18 chromatography (water/acetonitrile gradient). Yield: 35 mg (43 μmol, 43%) colourless solid. UV (HPLC): *λ*_max_=265 nm; HPLC: *t*_R_=11.83 min (method A); 10.09 min (method B); ^1^H-NMR (200 MHz, CD_3_OD): *δ* 7.63 (*d*, *J*=1.3 Hz, 1H), 7.44–7.32 (*m*, 4H), 7.09–6.99 (*m*, 4H), 6.90 (dt, *J*=3.4 Hz, *J*=1.6 Hz, 1H), 6.43 (dt, *J*=5.9 Hz, *J*=1.7 Hz, 1H), 5.77 (ddd, *J*=5.9 Hz, *J*=2.4 Hz, *J*=1.4 Hz, 1H), 5.12 (*d*, *J*=8.0 Hz, 4H), 4.94–4.89 (*m*, 1H), 4.31–4.08 (*m*, 2H), 2.58 (*q*, *J*=7.5 Hz, 4H), 1.86 (*d*, *J*=1.3 Hz, 3H), 1.20 (*t*, *J*=7.5 Hz, 6H); ^13^C-NMR (75 MHz, CD_3_OD): *δ* 174.5, 166.5, 152.8, 152.4, 138.7, 135.7, 134.9 (*d*, *J*=7.6 Hz), 130.4 (*d*, *J*=2.4 Hz), 127.2, 122.9, 112.0, 90.8, 87.2 (*d*, *J*=9.0 Hz), 70.3 (*d*, *J*=5.6 Hz), 67.6 (*d*, *J*=6.3 Hz), 28.3, 12.5, 9.3; ^31^P-NMR (81 MHz, CD_3_OD): *δ* −11.76 (*d*, *J*=19.4 Hz), −13.19 (*d*, *J*=17.1 Hz), −23.51 (*t*, *J*=18.4 Hz); IR: 3,195, 2,987, 1,758, 1,691, 1,254 cm^−1^; MALDI-MS (*m/z*): [M-H]^−^ calcd. for C_30_H_35_N_2_O_17_P_3_, 787.108; found, 787.275.

### γ-Bis-(4-pentanoyloxybenzyl)-d4TTP **3c** (ammonium salt)

General procedure D with 90 mg d4TDP **6** (0.10 mmol, 1.0 eq.), 0.11 g **5c** (0.21 mmol, 2.0 eq.), 0.50 ml 0.25 M solution of DCI in acetonitrile (0.13 mmol, 1.2 eq.), 40 μl 5.5 M solution of *t*-BuOOH in *n*-decane (0.21 mmol, 2.1 eq.) in 0.5 ml acetonitrile. The crude product was purified using automatic RP-18 chromatography (water/acetonitrile gradient). Yield: 43 mg (49 μmol, 47%) colourless solid. UV (HPLC): *λ*_max_=265 nm; HPLC: *t*_R_=13.61 min (method A); ^1^H-NMR (400 MHz, CD_3_OD): *δ* 7.59 (*d*, *J*=1.5 Hz, 1H), 7.38–7.32 (*m*, 4H), 7.04–6.97 (*m*, 4H), 6.87 (dt, *J*=3.4 Hz, *J*=1.7 Hz, 1H), 6.39 (dt, *J*=5.9 Hz, *J*=1.8 Hz, 1H), 5.77 (ddd, *J*=5.9 Hz, *J*=2.4 Hz, *J*=1.4 Hz, 1H), 5.12 (*d*, *J*=8.1 Hz, 4H), 4.94–4.84 (*m*, 1H), 4.26–4.08 (*m*, 2H), 2.57–2.50 (*m*, 4H), 1.84 (*d*, *J*=1.5 Hz, 3H), 1.71–1.61 (*m*, 4H), 1.47–1.26 (*m*, 4H), 0.93 (*t*, *J*=7.4 Hz, 6H); ^13^C-NMR (101 MHz, CD_3_OD): *δ* 173.8, 166.5, 152.7, 152.3, 138.6, 135.6, 134.8 (*d*, *J*=7.3 Hz), 130.5 (*d*, *J*=3.1 Hz), 127.2, 122.9, 112.0, 90.8, 87.1 (*d*, *J*=8.8 Hz), 70.4 (dd, *J*=6.0 Hz, *J*=2.1 Hz), 67.9 (*d*, *J*=5.4 Hz), 34.7, 28.1, 23.2, 14.1, 12.5; ^31^P-NMR (81 MHz, CD_3_OD): *δ* −11.76 (*d*, *J*=19.3 Hz), −13.19 (*d*, *J*=17.1 Hz), −23.51 (*t*, *J*=18.2 Hz); IR: 3,183, 2,959, 1,755, 1,687, 1,219 cm^−1^; MALDI-MS (*m/z*): [M-H]^−^ calcd. for C_34_H_43_N_2_O_17_P_3_, 843.170; found, 843.267.

### γ-Bis-(4-heptanoyloxybenzyl)-d4TTP **3d** (ammonium salt)

General procedure D with 99 mg d4TDP **6** (0.11 mmol, 1.0 eq.), 0.14 g **5d** (0.23 mmol, 2.0 eq.), 0.55 ml 0.25 M solution of DCI in acetonitrile (0.14 mmol, 1.2 eq.), 44 μl 5.5 M solution of *t*-BuOOH in *n*-decane (0.24 mmol, 2.1 eq.) in 0.5 ml acetonitrile. The crude product was purified using automatic RP-18 chromatography (water/acetonitrile gradient). Yield: 41 mg (44 μmol, 40%) colourless solid. UV (HPLC): *λ*_max_=265 nm; HPLC: *t*_R_=15.40 min (method A); ^1^H-NMR (400 MHz, CD_3_OD): *δ* 7.68 (*d*, *J*=1.2 Hz, 1H), 7.46–7.39 (*m*, 4H), 7.11–7.05 (*m*, 4H), 6.95 (dt, *J*=3.4 Hz, *J*=1.6 Hz, 1H), 6.48 (dt, *J*=6.1 Hz, *J*=1.7 Hz, 1H), 5.86–5.80 (*m*, 1H), 5.18 (*d*, *J*=8.2 Hz, 4H), 5.01–4.94 (*m*, 1H), 4.34–4.16 (*m*, 2H), 2.60 (*t*, *J*=7.4 Hz, 4H), 1.92 (*d*, *J*=1.2 Hz, 3H), 1.76 (quint, *J*=7.4 Hz, 4H), 1.51–1.34 (*m*, 12H), 0.96 (*t*, *J*=6.8 Hz, 6H); ^13^C-NMR (101 MHz, CD_3_OD): *δ* 173.8, 166.5, 152.8, 152.3, 138.4, 135.8, 134.9 (*d*, *J*=7.8 Hz), 130.5 (*d*, *J*=2.9 Hz), 127.2, 122.9, 112.1, 90.8, 87.2 (*d*, *J*=8.9 Hz), 70.4 (*d*, *J*=6.8 Hz), 67.9 (*d*, *J*=4.9 Hz), 35.0, 32.7, 29.9, 23.6, 25.9, 14.4, 12.5; ^31^P-NMR (162 MHz, CD_3_OD): *δ* −11.64 (br.s), −13.08 (*d*, *J*=17.5 Hz), −23.47 (br.s); IR: 3,190, 2,928, 1,756, 1,689, 1,250 cm^−1^; MALDI-MS (*m/z*): [M-H]^−^ calcd. for C_38_H_51_N_2_O_17_P_3_, 899.233; found, 899.229.

### γ-Bis-(4-nonanoyloxybenzyl)-d4TTP **3e** (ammonium salt)

General procedure D with 0.15 g d4TDP **6** (0.17 mmol, 1.0 eq.), 0.22 g **5e** (0.34 mmol, 2.0 eq.), 0.88 ml 0.25 M solution of DCI in acetonitrile (0.22 mmol, 1.3 eq.), 68 μl 5.5 M solution of *t*-BuOOH in *n*-decane (0.37 mmol, 2.2 eq.) in 3 ml acetonitrile. The crude product was purified using automatic RP-18 chromatography (water/acetonitrile gradient). Yield: 70 mg (71 μmol, 42%) beige solid. UV (HPLC): *λ*_max_=265 nm; HPLC: *t*_R_=17.31 min (method A); ^1^H-NMR (300 MHz, CD_3_OD): *δ* 7.65 (*d*, *J*=1.3 Hz, 1H), 7.42–7.35 (*m*, 4H), 7.07–7.00 (*m*, 4H), 6.92 (dt, *J*=3.5 Hz, *J*=1.6 Hz, 1H), 6.45 (dt, *J*=6.1 Hz, *J*=1.7 Hz, 1H), 5.79 (ddd, *J*=6.0 Hz, *J*=2.4 Hz, *J*=1.7 Hz, 1H), 5.14 (*d*, *J*=8.1 Hz, 4H), 4.96–4.90 (*m*, 1H), 4.31–4.13 (*m*, 2H), 2.57 (*t*, *J*=7.4 Hz, 4H), 1.88 (*d*, *J*=1.3 Hz, 3H), 1.72 (quint, *J*=7.3 Hz, 4H), 1.49–1.24 (*m*, 20H), 0.96–0.85 (*m*, 6H); ^13^C-NMR (75 MHz, CD_3_OD): *δ* 173.4, 166.5, 152.9, 152.3, 138.4, 135.8, 134.5, 130.2 (*d*, *J*=2.4 Hz), 126.9, 122.6, 111.9, 90.5, 86.8 (*d*, *J*=9.5 Hz), 70.1 (*d*, *J*=5.2 Hz), 67.6 (*d*, *J*=5.6 Hz), 34.7, 32.7, 30.1, 30.1, 29.9, 25.7, 23.5, 14.2, 12.2; ^31^P-NMR (162 MHz, CD_3_OD): *δ* −11.83 (*d*, *J*=20.1 Hz), −3.28 (*d*, *J*=17.5 Hz), −23.82 (*t*, *J*=18.8 Hz); IR: 3,192, 3,062, 2,926, 1,757, 1,694, 1,250 cm^−1^; MALDI-MS (*m/z*): [M-H]^−^ calcd. for C_42_H_59_N_2_O_17_P_3_, 955.295; found, 955.296.

### γ-Bis-(4-decanoyloxybenzyl)-d4TTP **3f**

General procedure D with 71 mg d4TDP **6** (82 μmol, 1.0 eq.), 0.11 g **5f** (0.16 mmol, 2.0 eq.), 0.40 ml 0.25 M solution of DCI in acetonitrile (0.10 mmol, 1.2 eq.), 32 μl 5.5 M solution of *t*-BuOOH in *n*-decane (0.18 mmol, 2.2 eq.) in 1.2 ml acetonitrile. The crude product was purified using automatic RP-18 chromatography (water/acetonitrile gradient). Yield: 23 mg (21 μmol, 26%) colourless solid (counterions: 0.2 × Bu_4_N^+^, 1.8 × NH_4_^+^). UV (HPLC): *λ*_max_=265 nm; HPLC: *t*_R_=18.06 min (method A); ^1^H-NMR (400 MHz, CD_3_OD): *δ* 7.69 (*d*, *J*=1.4 Hz, 1H), 7.46–7.39 (*m*, 4H), 7.12–7.05 (*m*, 4H), 6.96 (dt, *J*=3.4 Hz, *J*=1.6 Hz, 1H), 6.50 (dt, *J*=6.0 Hz, *J*=1.7 Hz, 1H), 5.86–5.81 (*m*, 1H), 5.19 (*d*, *J*=8.1 Hz, 4H), 5.00–4.95 (*m*, 1H), 4.35–4.17 (*m*, 2H), 3.30–3.23 (*m*, 1.5H), 2.61 (*t*, *J*=7.4 Hz, 4H), 1.93 (*d*, *J*=1.4 Hz, 3H), 1.76 (quint, *J*=7.3 Hz, 4H), 1.73–1.64 (*m*, 1.5H), 1.52–1.28 (*m*, 25.5H), 1.06 (*t*, *J*=7.4 Hz, 2.3H), 0.97–0.90 (*m*, 6H); ^13^C-NMR (101 MHz, CD_3_OD): *δ* 173.7, 166.7, 152.6, 152.6, 138.7, 135.8, 135.0 (*d*, *J*=7.8 Hz), 130.5 (*d*, *J*=2.9 Hz), 127.1, 122.9, 112.1, 90.9, 87.2 (*d*, *J*=9.7 Hz), 70.4 (dd, *J*=5.6 Hz, *J*=1.7 Hz), 67.9 (*d*, *J*=4.8 Hz), 59.5, 35.0, 33.0, 30.6, 30.4, 30.4, 30.2, 26.0, 23.7, 24.8, 19.4, 14.4, 13.9, 12.5; ^31^P-NMR (162 MHz, CD_3_OD): *δ* −11.80 (*d*, *J*=19.9 Hz), −13.07 (*d*, *J*=17.5 Hz), −23.82 (br.s); IR: 3,174, 2,925, 1,758, 1,690, 1,249 cm^−1^; MALDI-MS (*m/z*): [M-H]^−^ calcd. for C_44_H_63_N_2_O_17_P_3_, 983.327; found, 983.512.

### γ-Bis-(4-dodecanoyloxybenzyl)-d4TTP **3g**

General procedure D with 74 mg d4TDP **6** (85 μmol, 1.0 eq.), 0.13 g **5g** (0.17 mmol, 2.0 eq.), 0.41 ml 0.25 M solution of DCI in acetonitrile (0.11 mmol, 1.2 eq.), 33 μl 5.5 M solution of *t*-BuOOH in *n*-decane (0.18 mmol, 2.1 eq.) in 1.2 ml acetonitrile and 1.0 ml THF. The crude product was purified by automatic RP-18 chromatography (water/acetonitrile gradient). Yield: 44 mg (37 μmol, 44%) colourless solid (counterions: 1.6 × Bu_4_N^+^, 0.4 × NH_4_^+^). UV (HPLC): *λ*_max_=265 nm; HPLC: *t*_R_=20.15 min (method A); ^1^H-NMR (400 MHz, CD_3_OD): *δ* 7.74 (*d*, *J*=1.5 Hz, 1H), 7.47–7.39 (*m*, 4H), 7.10–7.03 (*m*, 4H), 6.96 (dt, *J*=3.4 Hz, *J*=1.6 Hz, 1H), 6.56 (dt, *J*=6.0 Hz, *J*=1.8 Hz, 1H), 5.83–5.78 (*m*, 1H), 5.22 (dd, *J*=8.0 Hz, *J*=2.0 Hz, 4H), 5.01–4.95 (*m*, 1H), 4.43–4.19 (*m*, 2H), 3.31–3.19 (*m*, 12.8H), 2.61 (*t*, *J*=7.4 Hz, 4H), 1.93 (*d*, *J*=1.5 Hz, 3H), 1.76 (quint, *J*=6.7 Hz, 4H), 1.73–1.63 (*m*, 12.8H), 1.44 (sext, *J*=7.4 Hz, 12.8H), 1.51–1.28 (*m*, 32H), 1.05 (*t*, *J*=7.4 Hz, 19.2H), 0.98–0.89 (*m*, 6H); ^13^C-NMR (101 MHz, CD_3_OD): *δ* 173.7, 166.5, 152.8, 152.2, 138.8, 136.2, 135.2 (*d*, *J*=7.8 Hz), 130.5 (*d*, *J*=2.8 Hz), 126.9, 122.8, 112.1, 90.8, 87.4 (*d*, *J*=6.3 Hz), 70.4 (*d*, *J*=5.7 Hz), 67.8, 59.4, 35.0, 33.1, 30.7, 30.7, 30.6, 30.4, 30.4, 30.2, 26.0, 23.7, 24.8, 20.7, 14.5, 14.0, 12.5; ^31^P-NMR (162 MHz, CD_3_OD): *δ* −11.91 (br.s), −13.28 (*d*, *J*=17.6 Hz), −3.99 (br.s); IR: 3,203, 2,925, 1,757, 1,690, 1,262 cm^−1^; MALDI-MS (*m/z*): [M-H]^−^ calcd. for C_48_H_69_N_2_O_17_P_3_, 1039.389; found, 1039.561.

### γ-Bis-(4-tetradecanoyloxybenzyl)-d4TTP **3h**

General procedure D with 48 mg d4TDP **6** (53 μmol, 1.0 eq.), 85 mg **5h** (0.11 mmol, 2.0 eq.), 0.28 ml 0.25 M solution of DCI in acetonitrile (69 μmol, 1.3 eq.), 21 μl 5.5 M solution of *t*-BuOOH in *n*-decane (0.12 mmol, 2.2 eq.) in 0.5 ml acetonitrile and 0.7 ml THF. The reaction was restarted once. The crude product was purified using automatic RP-18 chromatography (water/THF gradient). Yield: 72 mg (37 μmol, 70% (exclusive contamination)) colourless solid (counterions: 1.0 × Bu_4_N^+^, 1.0 × DIPAH^+^), contaminated with Bu_4_N^+^ and di*iso*propylammonium salts. UV (HPLC): *λ*_max_=265 nm; HPLC: *t*_R_=22.22 min (method A); ^1^H-NMR (300 MHz, THF-*d*_8_): *δ* 10.10 (*s*, 1H), 7.88 (*d*, *J*=1.2 Hz, 1H), 7.53–7.44 (*m*, 4H), 7.04–6.96 (*m*, 4H), 6.91 (dt, *J*=3.3 Hz, *J*=1.6 Hz, 1H), 6.62 (dt, *J*=5.9 Hz, *J*=1.7 Hz, 1H), 5.61–5.56 (*m*, 1H), 5.26–5.17 (*m*, 4H), 4.85–4.78 (*m*, 1H), 4.44–4.31 (*m*, 1H), 4.14–4.01 (*m*, 1H), 3.50–3.39 (*m*, 8H), 3.33–2.85 (*m*, 2H), 2.52 (*t*, *J*=7.5 Hz, 4H), 1.91 (*d*, *J*=1.2 Hz, 3H), 1.77–1.59 (*m*, 12H), 1.50–1.23 (*m*, 60H), 0.94 (*t*, *J*=7.4 Hz, 12H), 0.89 (*t*, *J*=6.8 Hz, 6H); ^13^C-NMR (75 MHz, THF-*d*_8_): *δ* 172.2, 164.9, 152.1, 151.8, 138.2, 136.9, 136.1 (*d*, *J*=8.5 Hz), 130.1 (*d*, *J*=1.6 Hz), 126.2, 122.3, 111.3, 90.1, 87.8 (*d*, *J*=8.5 Hz), 69.2 (*d*, *J*=5.4 Hz), 67.2, 59.3, 47.2, 34.9, 33.1, 30.8, 30.8, 30.8, 30.8, 30.7, 30.5, 30.5, 30.2, 26.0, 25.0, 23.8, 20.7, 19.9, 14.6, 14.4, 12.8; ^31^P-NMR (162 MHz, THF-*d*_8_): *δ* −14.16 (*d*, *J*=20.8 Hz), −14.65 (*d*, *J*=17.8 Hz), −23.82 (*t*, *J*=19.1 Hz); IR: 3,400, 2,924, 1,757, 1,689, 1,263 cm^−1^; MALDI-MS (*m/z*): [M-H]^−^ calcd. for C_52_H_79_N_2_O_17_P_3_, 1095.452; found, 1095.503.

### γ-Bis-(4-hexadecanoyloxybenzyl)-d4TTP **3i**

General procedure D with 90 mg d4TDP **6** (0.10 mmol, 1.0 eq.), 0.18 g **5i** (0.21 mmol, 2.0 eq.), 0.50 ml 0.25 M solution of DCI in acetonitrile (0.13 mmol, 1.3 eq.), 42 μl 5.5 M solution of *t*-BuOOH in *n*-decane (0.23 mmol, 2.2 eq.) in 0.3 ml acetonitrile and 0.9 ml THF. The reaction was restarted once. The crude product was purified using automatic RP-18 chromatography (water/THF gradient). Yield: 96 mg (64 μmol, 62%) colourless solid (counterions: 1.0 × Bu_4_N^+^, 1.0 × DIPAH^+^). UV (HPLC): *λ*_max_=265 nm; HPLC: *t*_R_=23.28 min (method A); ^1^H-NMR (500 MHz, THF-*d*_8_): *δ* 10.22 (*s*, 1H), 7.88 (*d*, *J*=1.3 Hz, 1H), 7.48–7.42 (*m*, 4H), 7.05–6.99 (*m*, 4H), 6.92 (dt, *J*=3.4 Hz, *J*=1.6 Hz, 1H), 6.55 (dt, *J*=5.8 Hz, *J*=1.6 Hz, 1H), 5.67–5.61 (*m*, 1H), 5.22–5.13 (*m*, 4H), 4.87–4.82 (*m*, 1H), 4.38–4.29 (*m*, 1H), 4.15–4.07 (*m*, 1H), 3.43–3.27 (*m*, 8H), 3.31–3.15 (*m*, 2H), 2.53 (*t*, *J*=7.5 Hz, 4H), 1.90 (*d*, *J*=1.0 Hz, 3H), 1.74–1.62 (*m*, 12H), 1.48–1.20 (*m*, 68H), 0.95 (*t*, *J*=7.2 Hz, 12H), 0.89 (*t*, *J*=6.8 Hzm, 6H); ^13^C-NMR (75 MHz, THF-*d*_8_): *δ* 172.1, 164.9, 152.1, 152.0, 137.8, 136.3, 135.5 (*d*, *J*=8.6 Hz), 130.1, 126.6, 122.5, 111.3, 90.2, 87.3 (*d*, *J*=7.7 Hz), 69.4 (*d*, *J*=7.2 Hz), 67.0, 59.3, 47.4, 34.9, 33.0, 30.8, 30.8, 30.8, 30.8, 30.7, 30.5, 30.5, 30.2, 26.1, 24.8, 23.6, 20.7, 19.8, 14.6, 14.4, 12.8; ^31^P-NMR (202 MHz, THF-*d*_8_): *δ* −12.56 (*d*, *J*=19.6 Hz), −13.38 (*d*, *J*=17.5 Hz), −24.17 (*t*, *J*=18.5 Hz); IR: 2,987, 2,916, 1,756, 1,691, 1,251 cm^−1^; MALDI-MS (*m/z*): [M-H]^−^ calcd. for C_56_H_85_N_2_O_17_P_3_, 1151.515; found, 1151.663.

### γ-Bis-(4-octadecanoyloxybenzyl)-d4TTP **3j**

General procedure D with 87 mg d4TDP **6** (0.10 mmol, 1.0 eq.), 0.19 g **5j** (0.20 mmol, 2.0 eq.), 0.52 ml 0.25 M solution of DCI in acetonitrile (0.13 mmol, 1.3 eq.), 38 μl 5.5 M solution of *t*-BuOOH in *n*-decane (0.21 mmol, 2.1 eq.) in 0.3 ml acetonitrile and 0.9 ml THF. The reaction was restarted once. The crude product was purified using automatic RP-18 chromatography (water/THF gradient). Yield: 69 mg (44 μmol, 44%) colourless solid (counterions: 1.0 × Bu_4_N^+^, 1.0 × DIPAH^+^).

UV (HPLC): *λ*_max_=265 nm; HPLC: *t*_R_=19.45 min (method C); ^1^H-NMR (300 MHz, THF-*d*_8_): *δ* 10.16 (*s*, 1H), 7.84 (*d*, *J*=1.4 Hz, 1H), 7.50–7.41 (*m*, 4H), 7.05–6.96 (*m*, 4H), 6.91 (dt, *J*=3.3 Hz, *J*=1.5 Hz, 1H), 6.62–6.53 (*m*, 1H), 5.65–5.57 (*m*, 1H), 5.24–5.13 (*m*, 4H), 4.89–4.79 (*m*, 1H), 4.43–4.30 (*m*, 1H), 4.17–4.04 (*m*, 1H), 3.51–3.28 (*m*, 8H), 3.30–3.09 (*m*, 2H), 2.51 (*t*, *J*=7.4 Hz, 4H), 1.91 (*d*, *J*=0.7 Hz, 3H), 1.77–1.60 (*m*, 13H), 1.49–1.19 (*m*, 76H), 1.00–0.82 (*m*, 18H); ^13^C-NMR (75 MHz, THF-*d*_8_): *δ* 172.1, 164.8, 152.1, 151.9, 138.0, 136.6, 135.8 (*d*, *J*=7.8 Hz), 130.1, 126.5, 122.4, 111.3, 90.2, 87.5 (*d*, *J*=8.4 Hz), 69.2 (*d*, *J*=5.3 Hz), 67.1, 59.3, 47.2, 34.9, 33.1, 30.8, 30.8, 30.7, 30.5, 30.5, 30.3, 26.0, 24.9, 23.8, 20.8, 19.8, 14.6, 14.4, 12.8; ^31^P-NMR (162 MHz, THF-*d*_8_): *δ* −12.74 (*d*, *J*=19.2 Hz), −12.83 (*d*, *J*=18.3 Hz), −23.46 (*t*, *J*=18.7 Hz); IR: 2,959, 2,916, 1,756, 1,688, 1,252 cm^−1^; MALDI-MS (*m/z*): [M-H]^−^ calcd. for C_60_H_95_N_2_O_17_P_3_, 1207.577; found, 1207.670.

### γ-Bis(4-(*Z*)-octadec-9-enoyloxybenzyl)-d4TTP **3k**

General procedure D with 96 mg d4TDP **6** (0.11 mmol, 1.0 eq.), 0.20 g **5k** (0.22 mmol, 2.0 eq.), 0.53 ml 0.25 M solution of DCI in acetonitrile (0.13 mmol, 1.2 eq.), 42 μl 5.5 M solution of *t*-BuOOH in *n*-decane (0.23 mmol, 2.1 eq.) in 0.3 ml acetonitrile and 0.9 ml THF. The reaction was restarted once. The crude product was purified using automatic RP-18 chromatography (water/THF gradient). Yield: 99 mg (64 μmol, 58%) colourless solid (counterions: 1.0 × Bu_4_N^+^, 1.0 × DIPAH^+^). UV (HPLC): *λ*_max_=265 nm; HPLC: *t*_R_=22.92 min (method A); ^1^H-NMR (300 MHz, THF-*d*_8_): *δ* 10.20 (*s*, 1H), 7.86 (*s*, 1H), 7.52–7.42 (*m*, 4H), 7.07–6.96 (*m*, 4H), 6.94–6.88 (*m*, 1H), 6.67–6.53 (*m*, 1H), 5.68–5.54 (*m*, 1H), 5.44–5.27 (*m*, 4H), 5.24–5.14 (*m*, 4H), 4.90–4.79 (*m*, 1H), 4.43–4.31 (*m*, 1H), 4.18–4.03 (*m*, 1H), 3.57–3.25 (*m*, 8H), 3.32–3.02 (*m*, 2H), 2.52 (*t*, *J*=7.5 Hz, 4H), 2.14–1.95 (*m*, 8H), 1.91 (*s*, 3H), 1.80–1.58 (*m*, 12H), 1.54–1.16 (*m*, 60H), 1.05–0.75 (*m*, 18H); ^13^C-NMR (75 MHz, THF-*d*_8_): *δ* 172.1, 164.8, 152.1, 151.9, 138.0, 137.3, 135.9 (*d*, *J*=7.9 Hz), 130.7, 130.7, 130.1 (*d*, *J*=1.5 Hz), 126.3, 122.4, 111.2, 90.2, 87.6 (*d*, *J*=8.8 Hz), 69.1, 67.1, 59.3, 47.2, 34.9, 33.0, 30.9, 30.7, 30.5, 30.4, 30.3, 30.2, 28.2, 28.2, 25.9, 25.0, 23.7, 20.8, 19.8, 14.7, 14.4, 12.8; ^31^P-NMR (162 MHz, THF-*d*_8_): *δ* −14.44 (*d*, *J*=19.0 Hz), −14.94 (*d*, *J*=18.0 Hz), −25.55 (*t*, *J*=18.5 Hz); IR: 3,358, 2,965, 2,924, 1,757, 1,689, 1,262 cm^−1^; MALDI-MS (*m/z*): [M-H]^−^ calcd. for C_60_H_91_N_2_O_17_P_3_, 1203.557; found, 1203.546.

### γ-Bis-(4-methyloxycarbonyloxybenzyl)-d4TTP **3l** (ammonium salt)

General procedure D with 99 mg d4TDP **6** (0.11 mmol, 1.0 eq.), 0.11 g **5l** (0.23 mmol, 2.0 eq.), 0.59 ml 0.25 M solution of DCI in acetonitrile (0.15 mmol, 1.3 eq.), 46 μl 5.5 M solution of *t*-BuOOH in *n*-decane (0.25 mmol, 2.2 eq.) in 0.7 ml acetonitrile. The crude product was purified using automatic RP-18 chromatography (water/acetonitrile gradient). Yield: 49 mg (59 μmol, 52%) colourless solid. UV (HPLC): *λ*_max_=265 nm; HPLC: *t*_R_=11.28 min (method A); 9.17 min (method B); ^1^H-NMR (400 MHz, CD_3_OD): *δ* 7.68 (*d*, *J*=1.2 Hz, 1H), 7.43–7.38 (*m*, 4H), 7.16–7.11 (*m*, 4H), 6.92 (dt, *J*=3.4 Hz, *J*=1.8 Hz, 1H), 6.47 (dt, *J*=6.0 Hz, *J*=1.7 Hz, 1H), 5.82 (ddd, *J*=6.1 Hz, *J*=2.5 Hz, *J*=1.4 Hz, 1H), 5.18 (*d*, *J*=8.0 Hz, 4H), 4.96–4.90 (*m*, 1H), 4.31–4.15 (*m*, 2H), 3.86 (*s*, 6H), 1.88 (*d*, *J*=1.2 Hz, 3H); ^13^C-NMR (101 MHz, CD_3_OD): *δ* 166.7, 155.7, 152.8, 152.7, 138.7, 135.8, 135.4 (*d*, *J*=7.6 Hz), 130.5 (*d*, *J*=3.5 Hz), 127.1, 122.3, 112.1, 90.8, 87.3 (*d*, *J*=9.5 Hz), 70.3 (dd, *J*=5.8 Hz, *J*=2.1 Hz), 67.6 (*d*, *J*=5.9 Hz), 56.0, 12.5; ^31^P-NMR (162 MHz, CD_3_OD): *δ* −11.79 (*d*, *J*=19.8 Hz), −13.23 (*d*, *J*=17.6 Hz), −23.71 (*t*, *J*=18.5 Hz); IR: 3,191, 3,050, 1,764, 1,692, 1,263 cm^−1^; MALDI-MS (*m/z*): [M-H]^−^ calcd. for C_28_H_31_N_2_O_19_P_3_, 791.066; found, 791.003.

### γ-Bis-(4-octyloxycarbonyloxybenzyl)-d4TTP **3m**

General procedure D with 0.11 g d4TDP **6** (0.13 mmol, 1.0 eq.), 0.18 g **5m** (0.26 mmol, 2.0 eq.), 0.68 ml 0.25 M solution of DCI in acetonitrile (0.17 mmol, 1.3 eq.), 53 μl 5.5 M solution of *t*-BuOOH in *n*-decane (0.29 mmol, 2.2 eq.) in 0.7 ml acetonitrile. The crude product was purified using automatic RP-18 chromatography (water/acetonitrile gradient). Yield: 75 mg (69 μmol, 52%) colourless solid (counterions: 0.3 × Bu_4_N^+^, 1.7 × NH_4_^+^). UV (HPLC): *λ*_max_=265 nm; HPLC: *t*_R_=16.72 min (method A); ^1^H-NMR (400 MHz, CD_3_OD): *δ* 7.65 (d, *J*=1.1 Hz, 1H), 7.43MALDI-MS (*m/z*): [M-H]^−^ calcd7.36 (*m*, 4H), 7.17MALDI-MS (*m/z*): [M-H]^−^ calcd7.09 (*m*, 4H), 6.91 (dt, *J*=3.5 Hz, *J*=1.9 Hz, 1H), 6.44 (dt, *J*=6.0 Hz, *J*=1.8 Hz, 1H), 5.82 (ddd, *J*=6.0 Hz, *J*=2.4 Hz, *J*=1.3 Hz, 1H), 5.14 (d, *J*=8.2 Hz, 4H), 4.96–4.90 (*m*, 1H), 4.29–4.13 (*m*, 2H), 4.22 (*t*, *J*=6.6 Hz, 4H), 3.25–3.15 (*m*, 2.5H), 1.88 (d, *J*=1.1 Hz, 3H), 1.77–1.64 (*m*, 4H), 1.67–1.57 (*m*, 2.5H), 1.47–1.22 (*m*, 22.5H), 1.00 (*t*, *J*=7.3 Hz, 3.8H), 0.90 (*t*, *J*=6.7 Hz, 6H); ^13^C-NMR (101 MHz, CD_3_OD): *δ* 166.5, 155.1, 152.7, 152.7, 138.6, 135.7, 135.2 (d, *J*=7.6 Hz), 130.5 (d, *J*=2.3 Hz), 127.2, 122.3, 112.0, 90.8, 87.3 (d, *J*=9.5 Hz), 70.3 (dd, *J*=5.4 Hz, *J*=1.4 Hz), 70.0, 67.8 (d, *J*=5.6 Hz), 59.4, 32.9, 30.3, 30.3, 29.7, 26.8, 24.7, 23.7, 20.7, 14.5, 14.0, 12.5; ^31^P-NMR (162 MHz, CD_3_OD): *δ* −11.68 (d, *J*=19.3 Hz), −13.15 (d, *J*=16.1 Hz), −23.53 (*t*, *J*=18.0 Hz); IR: 3,198, 2,926, 1,760, 1,690, 1,250 cm^−1^; MALDI-MS (*m/z*): [M-H]^−^ calcd. for C_42_H_59_N_2_O_19_P_3_, 987.285; found, 987.396.

### γ-Bis-(4-dodecyloxycarbonyloxybenzyl)-d4TTP **3n**

General procedure D with 80 mg d4TDP **6** (92 μmol, 1.0 eq.), 0.15 g **5n** (0.19 mmol, 2.0 eq.), 0.48 ml 0.25 M solution of DCI in acetonitrile (0.12 mmol, 1.3 eq.), 37 μl 5.5 M solution of t-BuOOH in *n*-decane (0.20 mmol, 2.1 eq.) in 0.5 ml acetonitrile and 0.5 ml THF. The crude product was purified using automatic RP-18 chromatography (water/acetonitrile gradient). Yield: 52 mg (38 μmol, 41%) colourless solid (counterions: 1.1 × Bu_4_N^+^, 0.9 × NH_4_^+^). UV (HPLC): *λ*_max_=265 nm; HPLC: *t*_R_=20.53 min (method A); ^1^H-NMR (300 MHz, CD_3_OD): *δ* 7.70 (*d*, *J*=1.3 Hz, 1H), 7.43–7.36 (*m*, 4H), 7.19–7.11 (*m*, 4H), 6.94 (dt, *J*=3.4 Hz, *J*=1.7 Hz, 1H), 6.52 (dt, *J*=6.0 Hz, *J*=1.8 Hz, 1H), 5.80 (ddd, *J*=6.0 Hz, *J*=2.4 Hz, *J*=1.4 Hz, 1H), 5.19 (*d*, *J*=8.0 Hz, 4H), 5.00–4.93 (*m*, 1H), 4.37–4.15 (*m*, 2H), 4.25 (*t*, *J*=6.6 Hz, 4H), 3.29–3.14 (*m*, 9H), 1.92 (*d*, *J*=1.3 Hz, 3H), 1.80–1.66 (*m*, 4H), 1.71–1.58 (*m*, 9H), 1.50–1.25 (*m*, 45H), 1.03 (*t*, *J*=7.4 Hz, 13.5H), 0.92 (*t*, *J*=6.6 Hz, 6H); ^13^C-NMR (75 MHz, CD_3_OD): *δ* 166.5, 155.1, 152.7, 152.6, 138.7, 136.0, 135.4 (*d*, *J*=7.8 Hz), 130.5 (*d*, *J*=2.4 Hz), 127.0, 122.3, 112.1, 90.8, 87.3 (*d*, *J*=9.2 Hz), 70.2 (*d*, *J*=5.6 Hz), 70.0, 67.8 (*d*, *J*=6.0 Hz), 59.5, 33.1, 30.8, 30.7, 30.6, 30.5, 30.3, 29.7, 26.8, 24.8, 23.7, 20.7, 19.4, 14.5, 13.9, 12.5; ^31^P-NMR (162 MHz, CD_3_OD): *δ* −12.07 (*d*, *J*=21.7 Hz), −13.38 (*d*, *J*=18.1 Hz), −24.20 (dd, *J*=21.7 Hz, *J*=18.1 Hz); IR: 2,923, 1,760, 1,689, 1,248 cm^−1^; MALDI-MS (*m/z*): [M-H]^−^ calcd. for C_50_H_75_N_2_O_19_P_3_, 1099.410; found, 1099.083.

### γ-Bis-(4-(butyl-ethane-1,2-diylbis(methylcarbamate))-oxybenzyl)-d4TTP **3o** (ammonium salt)

General procedure D with 75 mg d4TDP **6** (87 μmol, 1.0 eq.), 0.12 g **5o** (0.15 mmol, 1.7 eq.), 0.45 ml 0.25 M solution of DCI in acetonitrile (0.11 mmol, 1.3 eq.), 32 μl 5.5 M solution of *t*-BuOOH in *n*-decane (0.18 mmol, 2.1 eq.) in 1.5 ml acetonitrile. The crude product was purified using automatic RP-18 chromatography (water/acetonitrile gradient). Yield: 68 mg (60 μmol, 69%) colourless solid. UV (HPLC): *λ*_max_=265 nm; HPLC: *t*_R_=12.88 min (method A); ^1^H-NMR (400 MHz, CD_3_OD): *δ* 7.68 (*d*, *J*=1.0 Hz, 1H), 7.48–7.42 (*m*, 4H), 7.15–7.09 (*m*, 4H, rotamers), 6.96 (dt, *J*=3.5 Hz, *J*=1.7 Hz, 1H), 6.49 (dt, *J*=6.1 Hz, *J*=1.7 Hz, 1H), 5.86–5.82 (*m*, 1H), 5.22–5.16 (*m*, 4H), 5.00–4.96 (*m*, 1H), 4.34–4.19 (*m*, 2H), 4.17–4.05 (*m*, 4H), 3.71–3.53 (*m*, 8H, rotamers), 3.15–2.96 (*m*, 12H), 1.93 (*d*, *J*=1.0 Hz, 3H), 1.72–1.56 (*m*, 4H, rotamers), 1.50–1.34 (*m*, 4H, rotamers), 0.96, 0.90 (*t*, *J*=7.4 Hz, 6H, rotamers); ^13^C-NMR (101 MHz, CD_3_OD): *δ* 166.5, 158.3, 156.4, 152.8, 152.7, 138.6, 135.7, 134.6, 130.4 (*d*, *J*=2.7 Hz), 127.2, 123.1+122.9 (rotamers), 112.0 (C-5), 90.8 (C-1′), 87.2 (*d*, *J*=9.1 Hz), 70.4 (dd, *J*=5.4 Hz, *J*=2.5 Hz), 67.9 (*d*, *J*=5.8 Hz), 66.8+66.6 (rotamers), 48.1+47.8 (rotamers), 47.6+47.3 (rotamers), 35.5+35.0 (rotamers), 32.2+32.2 (rotamers), 20.2, 14.1+14.1 (rotamers), 12.5; ^31^P-NMR (162 MHz, CD_3_OD): *δ* −11.72 (d, *J*=19.5 Hz), −13.16 (*d*, *J*=17.8 Hz), −23.55 (*t*, *J*=18.1 Hz); IR: 3,191, 1,959, 1,687, 1,204 cm^−1^; MALDI-MS (*m/z*): [M-H]^−^ calcd. for C_44_H_63_N_6_O_21_P_3_, 1103.319; found, 1103.383.

### γ-Bis-(4-(octyl-ethane-1,2-diylbis(methylcarbamate))-oxybenzyl)-d4TTP **3p** (ammonium salt)

General procedure D with 80 mg d4TDP **6** (92 μmol, 1.0 eq.), 0.15 g **5p** (0.16 mmol, 1.7 eq.), 0.48 ml 0.25 M solution of DCI in acetonitrile (0.12 mmol, 1.3 eq.), 37 μl 5.5 M solution of *t*-BuOOH in *n*-decane (0.20 mmol, 2.2 eq.) in 0.8 ml acetonitrile. The crude product was purified using automatic RP-18 chromatography (water/acetonitrile gradient). Yield: 73 mg (58 μmol, 63%) colourless solid. UV (HPLC): *λ*_max_=265 nm; HPLC: *t*_R_=15.98 min (method A); ^1^H-NMR (400 MHz, CD_3_OD): *δ* 7.69 (br.s, 1H), 7.47–7.42 (*m*, 4H), 7.15–7.08 (*m*, 4H, rotamers), 6.96 (dt, *J*=3.5 Hz, *J*=1.6 Hz, 1H), 6.49 (dt, *J*=6.0 Hz, *J*=1.7 Hz, 1H), 5.83 (dt, *J*=6.0 Hz, *J*=1.7 Hz, 1H), 5.22–5.16 (*m*, 4H), 5.00–4.96 (*m*, 1H), 4.34–4.19 (*m*, 2H), 4.15–4.05 (*m*, 4H), 3.72–3.53 (*m*, 8H, rotamers), 3.18–2.96 (*m*, 12H), 1.93 (*s*, 3H, H-7), 1.74–1.59 (*m*, 4H, rotamers), 1.47–1.22 (*m*, 20H), 0.92 (*t*, *J*=6.2 Hz, 6H); ^13^C-NMR (101 MHz, CD_3_OD): *δ* 166.3, 158.4, 156.5, 152.8, 152.8, 138.6, 135.7, 134.6, 130.4 (*d*, *J*=2.7 Hz), 127.2, 123.2+123.0 (rotamers), 112.0, 90.8, 87.2 (*d*, *J*=9.2 Hz), 70.5–70.3 (*m*), 67.9 (*d*, *J*=5.9 Hz), 67.1+66.9 (rotamers), 48.2+47.9 (rotamers), 47.6+47.3 (rotamers), 35.5+35.4 (rotamers), 35.2+35.0 (rotamers), 32.9, 30.3, 30.3, 30.2+30.1 (rotamers), 27.0, 23.7, 14.5, 12.5; ^31^P-NMR (162 MHz, CD_3_OD): *δ* −11.71 (*d*, *J*=19.2 Hz), −13.16 (*d*, *J*=16.7 Hz), −3.54 (br.s, *J*=17.2 Hz); IR: 3,190, 2,925, 0,693, 1,205 cm^−1^; MALDI-MS (*m/z*): [M-H]^−^ calcd. for C_52_H_79_N_6_O_21_P_3_, 1215.444; found, 1215.630.

### γ-Bis-(4-(dodecyl-ethane-1,2-diylbis(methylcarbamate))-oxybenzyl)-d4TTP **3q** (ammonium salt)

General procedure D with 78 mg d4TDP **6** (90 μmol, 1.0 eq.), 0.22 g **5q** (0.15 mmol, 1.7 eq.), 0.47 ml 0.25 M solution of DCI in acetonitrile (0.12 mmol, 1.3 eq.), 36 μl 5.5 M solution of *t*-BuOOH in *n*-decane (0.20 mmol, 2.2 eq.) in 3.0 ml acetonitrile. The crude product was purified using automatic RP-18 chromatography (water/acetonitrile gradient). Yield: 36 mg (26 μmol, 29%) colourless solid. UV (HPLC): *λ*_max_=265 nm; HPLC: *t*_R_=19.04 min (method A); ^1^H-NMR (400 MHz, CD_3_OD): *δ* 7.66 (br.s, 1H), 7.46–7.39 (*m*, 4H), 7.13–7.06 (*m*, 4H, rotamers), 6.95–6.92 (*m*, 1H), 6.47 (dt, *J*=6.1 Hz, *J*=1.7 Hz, 1H), 5.83–5.79 (*m*, 1H), 5.20–5.13 (*m*, 4H), 4.98–4.93 (*m*, 1H), 4.33–4.16 (*m*, 2H), 4.14–4.01 (*m*, 4H), 3.71–3.53 (*m*, 8H, rotamers), 3.17–2.93 (*m*, 12H), 1.91 (*s*, 3H), 1.72–1.55 (*m*, 4H, rotamers), 1.45–1.21 (*m*, 36H), 0.91 (*t*, *J*=6.6 Hz, 6H); ^13^C-NMR (101 MHz, CD_3_OD): *δ* 166.4, 158.3, 156.4, 152.8, 152.7, 138.6, 135.7, 134.7–134.4 (*m*), 130.4 (*d*, *J*=2.5 Hz), 127.2, 123.2+123.0 (rotamers), 112.5, 90.8, 87.2 (*d*, *J*=9.2 Hz), 70.4 (dd, *J*=5.3 Hz, *J*=2.2 Hz), 67.9 (*d*, *J*=5.0 Hz), 67.1+66.9 (rotamers), 48.2+47.9 (rotamers), 47.6+47.3 (rotamers), 35.5+35.4 (rotamers), 35.2+34.9 (rotamers), 33.1, 30.7, 30.7, 30.6, 30.5, 30.4, 30.3, 30.2+30.1 (rotamers), 27.0, 23.7, 14.5, 12.5; ^31^P-NMR (162 MHz, CD_3_OD): *δ* −11.74 (br.s), −13.10 (*d*, *J*=16.1 Hz), −23.51 (br.s); IR: 3,191, 2,922, 1,697, 1,205 cm^−1^; MALDI-MS (*m/z*): [M-H]^−^ calcd. for C_60_H_95_N_6_O_21_P_3_, 1327.569; found, 1327.607.

### General procedure E: preparation of 5-Nitro-*cyclo*Sal-(4-alkanoyloxybenzyl)-monophosphates **17**

Corresponding 4-alkanoyloxybenzyl alcohol **9** (1.0 eq.) and 2.2 eq. di*iso*propylethylamine were dissolved in acetonitrile or THF and cooled to −20 °C. After dropwise addition of 2.0 eq., 5-nitrosaligenylchlorophosphite **18**, dissolved in acetonitrile or THF, the reaction mixture was allowed to warm to rt. The solution was kept at this temperature for 2 h. For oxidation, oxone (4.0 eq.) dissolved in water was added. The mixture was stirred for 15 min and immediately extracted with ethyl acetate. The organic phase was dried over Na_2_SO_4_, filtered and the solvent was removed by evaporation. The crude products were purified using preparative TLC (chromatotron).

### 5-Nitro-*cyclo*Sal-(4-acetyloxybenzyl)-monophosphate **17a**

General procedure E with a solution of 0.11 g 4-(hydroxymethyl)phenylacetate **9a** (0.67 mmol, 1.0 eq.) and 0.25 ml di*iso*propylethylamine (0.19 g, 1.5 mmol, 2.2 eq.) dissolved in 12 ml acetonitrile, 0.31 g 5-nitrosaligenylchlorophosphite **18** (1.3 mmol, 2.0 eq.) dissolved in 15 ml acetonitrile. For oxidation 1.7 g oxone (2.7 mmol, 4.0 eq.) were used. The crude product was purified using preparative TLC (CH_2_Cl_2_/MeOH 19:1 v/v+0.1% HOAc). Yield: 0.12 g (0.31 mmol, 46%) yellowish oil. TLC (PE/EE 1:1 v/v+0.1% HOAc): *R*_f_=0.45; ^1^H-NMR (300 MHz, CDCl_3_): *δ* 8.15–8.05 (*m*, 1H), 7.99–7.94 (*m*, 1H), 7.38–7.29 (*m*, 2H), 7.08–6.95 (*m*, 3H), 5.42–5.27 (*m*, 2H), 5.18 (*d*, *J*=10.1 Hz, 2H), 2.25 (*s*, 3H); ^13^C-NMR (75 MHz, CDCl_3_): *δ* 172.2, 154.3 (*d*, *J*=6.8 Hz), 151.2, 143.8, 132.2 (*d*, *J*=5.6 Hz), 129.6, 125.4, 122.0, 121.4, 121.4, 119.7 (*d*, *J*=9.2 Hz), 70.2 (*d*, *J*=6.0 Hz), 67.9 (*d*, *J*=7.1 Hz), 21.0; ^31^P-NMR (162 MHz, CDCl_3_): *δ* −10.30; IR: 3,075, 1,753, 1,193 cm^−1^; HRMS (ESI^+^, *m/z*): [M+Na]^+^ calcd. for C_16_H_14_NO_8_P, 402.0349; found, 402.0306.

### 5-Nitro-*cyclo*Sal-(4-nonanoyloxybenzyl)-monophosphate **17e**

General procedure E with a solution of 0.39 g 4-(hydroxymethyl)phenylnonanoate **9e** (1.5 mmol, 1.0 eq.) and 0.55 ml di*iso*propylethylamine (0.42 g, 3.3 mmol, 2.2 eq.) dissolved in 10 ml acetonitrile, 0.69 g 5-nitrosaligenylchlorophosphite **18** (3.0 mmol, 2.0 eq.) dissolved in 20 ml acetonitrile. For oxidation, 3.6 g oxone (5.9 mmol, 4.0 eq.) was used. The crude product was purified using preparative TLC (CH_2_Cl_2_/MeOH 19:1 v/v+0.1% HOAc). Yield: 0.57 g (1.2 mmol, 80%) beige solid. TLC (PE/EE 1:1 v/v+0.1% HOAc): *R*_f_=0.66; ^1^H-NMR (300 MHz, CDCl_3_): *δ* 8.19–8.11 (*m*, 1H), 8.02–7.95 (*m*, 1H), 7.42–7.32 (*m*, 2H), 7.12–6.98 (*m*, 3H), 5.45–5.29 (*m*, 2H), 5.22 (*d*, *J*=10.3 Hz, 2H), 2.54 (*t*, *J*=7.5 Hz, 2H), 1.74 (quint, *J*=7.5 Hz, 2H), 1.46–1.17 (*m*, 10H), 0.87 (*t*, *J*=6.8 Hz, 3H); ^13^C-NMR (75 MHz, CDCl_3_): *δ* 172.2, 154.6 (*d*, *J*=6.9 Hz), 151.5, 143.9, 132.2 (*d*, *J*=5.7 Hz), 129.8, 125.6 (*d*, *J*=1.4 Hz), 122.3, 121.6, 121.4, 119.9 (*d*, *J*=9.1 Hz), 70.4 (*d*, *J*=5.6 Hz), 67.9 (*d*, *J*=6.8 Hz), 34.4, 31.9, 29.3, 29.2, 29.2, 24.9, 22.7, 14.2; ^31^P-NMR (81 MHz, CDCl_3_): *δ* −10.73; IR: 2,921, 2,852, 1,749 cm^−1^; HRMS (ESI^+^, *m/z*): [M+Na]^+^ calcd. for C_23_H_28_NO_8_P, 500.1445; found, 500.1469.

### 5-Nitro-*cyclo*Sal-(4-octadecanoyloxybenzyl)-monophosphate **17j**

General procedure E with a solution of 0.43 g 4-(hydroxymethyl)phenyloctadecanoate **9j** (1.1 mmol, 1.0 eq.) and 0.38 ml di*iso*propylethylamine (0.29 g, 2.2 mmol, 2.0 eq.) dissolved in 20 ml THF, 0.69 g 5-nitrosaligenylchlorophosphite **18** (1.7 mmol, 1.5 eq.) dissolved in 15 ml THF. For oxidation, 2.0 g oxone (3.3 mmol, 3.0 eq.) was used. The crude product was purified using preparative TLC (CH_2_Cl_2_/MeOH 30:1 v/v+0.1% HOAc). Yield: 0.58 g (0.96 mmol, 87%) yellowish solid. TLC (PE/EE 1:1 v/v+0.1% HOAc): *R*_f_=0.72; ^1^H-NMR (400 MHz, CDCl_3_): *δ* 8.19–8.14 (*m*, 1H), 8.00 (*d*, *J*=2.7 Hz, 1H), 7.41–7.35 (*m*, 2H), 7.10–7.02 (*m*, 3H), 5.43–5.29 (*m*, 2H), 5.23 (*d*, *J*=10.4 Hz, 2H), 2.55 (*t*, *J*=7.5 Hz, 2H), 1.74 (quint, *J*=7.5 Hz, 2H), 1.46–1.19 (*m*, 28H), 0.87 (*t*, *J*=6.8 Hz, 3H); ^13^C-NMR (75 MHz, CDCl_3_): *δ* 172.2, 154.6, 151.6, 144.0, 132.2 (*d*, *J*=5.5 Hz), 129.9, 125.7, 122.2, 121.6, 121.3, 120.0 (*d*, *J*=9.4 Hz), 70.5 (*d*, *J*=5.8 Hz), 68.1 (*d*, *J*=7.1 Hz), 34.5, 32.0, 29.8, 29.8, 29.7, 29.6, 29.5, 29.4, 29.2, 25.0, 22.8, 14.2; ^31^P-NMR (162 MHz, CDCl_3_): *δ* −10.30; IR: 2,955, 2,849, 1,746 cm^−1^; HRMS (ESI^+^, *m/z*): [M+Na]^+^ calcd. for C_32_H_46_NO_8_P, 626.2853; found, 626.2821.

### General procedure F: preparation of γ-mono(4-alkanoyloxybenzyl)-d4TTP **4**

d4TDP **6** (1.0 eq.) was co-evaporated with DMF and dried in vacuum for 2 h. Then, 2.0–2.5 eq. of the corresponding 5-nitro-*cyclo*Sal-(4-alkanoyloxybenzyl)-monophosphate **17** was dissolved in a minimum of DMF followed by a dropwise addition to the nucleotide **8** dissolved in DMF. The reaction was stirred at rt for 20 h, and the solvent was removed under reduced pressure. The residue was dissolved in CH_2_Cl_2_/ammonium acetate (1 M). The layers were separated and the aqueous layer was freeze-dried. The crude product thus obtained was purified using automatic RP-18 chromatography (water/acetonitrile gradient). Subsequently, the cations were exchanged to ammonium ions using Dowex 50WX8 (ammonium form) cation exchange resin followed by a second RP-18 chromatography.

### γ-Mono-(4-acetyloxybenzyl)-d4TTP **4a** (ammonium salt)

General procedure F with 70 mg d4TDP **6** (81 μmol, 1.0 eq.) in 1.0 ml DMF and 77 mg 5-nitro-*cyclo*Sal-(4-acetyloxybenzyl)-monophosphate **17a** (0.20 mmol, 2.5 eq.) in 0.5 ml DMF. Yield: 16 mg (24 μmol, 30%) colourless solid. UV (HPLC): *λ*_max_=265 nm; HPLC: *t*_R_=10.88 min (method A), 5.23 min (method B); ^1^H-NMR (400 MHz, CD_3_OD): *δ* 7.69 (*d*, *J*=1.2 Hz, 1H), 7.52–7.48 (*m*, 2H), 7.10–7.04 (*m*, 2H), 6.95 (dt, *J*=3.5 Hz, *J*=1.6 Hz, 1H), 6.53 (dt, *J*=6.0 Hz, *J*=1.7 Hz, 1H), 5.85 (ddd, *J*=6.1 Hz, *J*=2.4 Hz, *J*=1.7 Hz, 1H), 5.07 (*d*, *J*=6.2 Hz, 2H), 5.02–4.97 (*m*, 1H), 4.32–4.17 (*m*, 2H), 2.29 (*s*, 3H), 1.93 (*d*, *J*=1.2 Hz, 3H); ^13^C-NMR (101 MHz, CD_3_OD): *δ* 171.3, 166.7, 153.0, 151.7, 138.7, 137.4 (*d*, *J*=8.7 Hz), 135.9, 129.7, 127.0, 122.5, 112.0, 90.9, 87.3 (*d*, *J*=9.1 Hz), 68.2 (*d*, *J*=5.3 Hz), 67.8 (*d*, *J*=6.1 Hz), 20.9, 12.5; ^31^P-NMR (162 MHz, CD_3_OD): *δ* −10.95 (*d*, *J*=19.0 Hz), −11.27 (*d*, *J*=18.8 Hz), −22.04 (*t*, *J*=18.8 Hz); IR: 3,190, 2,988, 1,687, 1,663, 1,217 cm^−1^; MALDI-MS (*m/z*): [M-H]^−^ calcd. for C_19_H_23_N_2_O_15_P_3_, 611.024; found, 611.044.

### γ-Mono-(4-nonanoyloxybenzyl)-d4TTP **4e** (ammonium salt)

General procedure F with 57 mg d4TDP **6** (66 μmol, 1.0 eq.) in 1.0 ml DMF and 63 mg 5-nitro-*cyclo*Sal-(4-nonanoyloxybenzyl)-monophosphate **17e** (0.13 mmol, 2.0 eq.) in 0.5 ml DMF. Yield: 15 mg (20 μmol, 30%) colourless solid. UV (HPLC): *λ*_max_=265 nm; HPLC: *t*_R_=13.03 min (method A); ^1^H-NMR (400 MHz, CD_3_OD): *δ* 7.70 (*d*, *J*=1.4 Hz, 1H), 7.53–7.47 (*m*, 2H), 7.08–7.03 (*m*, 2H), 6.96 (dt, *J*=3.4 Hz, *J*=1.6 Hz, 1H), 6.53 (dt, *J*=5.9 Hz, *J*=1.7 Hz, 1H), 5.88–5.82 (*m*, 1H), 5.08 (*d*, *J*=6.1 Hz, 2H), 5.02–4.97 (*m*, 1H), 4.34–4.17 (*m*, 2H), 2.60 (*t*, *J*=7.4 Hz, 2H), 1.94 (*s*, 3H), 1.76 (quint, *J*=7.3 Hz, 2H), 1.50–1.29 (*m*, 10H), 0.94 (*t*, *J*=6.7 Hz, 3H); ^13^C-NMR (101 MHz, CD_3_OD): *δ* 173.9, 166.5, 152.8, 151.7, 138.7, 137.4, 135.9, 129.7, 127.0, 122.5, 112.0, 90.9, 87.3 (*d*, *J*=5.2 Hz), 68.2 (*d*, *J*=5.3 Hz), 67.8 (*d*, *J*=5.4 Hz), 35.0, 33.0, 30.4, 30.2, 30.2, 26.0, 23.7, 14.4, 12.5; ^31^P-NMR (162 MHz, CD_3_OD): *δ* −10.99 (*d*, *J*=19.5 Hz), −11.31 (*d*, *J*=18.8 Hz), −22.12 (*t*, *J*=18.7 Hz); IR: 3,258, 2,973, 1,691, 1,066 cm^−1^; MALDI-MS (*m/z*): [M-H]^−^ calcd. for C_26_H_37_N_2_O_15_P_3_, 709.133; found, 709.238.

### γ-Mono-(4-octadecanoyloxybenzyl)-d4TTP **4j** (ammonium salt)

General procedure F with 56 mg d4TDP **6** (65 μmol, 1.0 eq.) in 1.0 ml DMF and 98 mg 5-nitro-*cyclo*Sal-(4-octa-decanoyloxybenzyl)-monophosphate **17j** (0.16 mmol, 2.5 eq.) in 0.5 ml DMF. Yield: 15 mg (17 μmol, 26%) colourless solid. UV (HPLC): *λ*_max_=265 nm; HPLC: *t*_R_=14.79 min (method A); ^1^H-NMR (400 MHz, CD_3_OD): *δ* 7.70 (*d*, *J*=1.3 Hz, 1H), 7.55–7.47 (*m*, 2H), 7.10–7.02 (*m*, 2H), 6.96 (dt, *J*=3.4 Hz, *J*=1.6 Hz, 1H), 6.53 (dt, *J*=6.0 Hz, *J*=1.7 Hz, 1H), 5.87–5.83 (*m*, 1H), 5.09 (*d*, *J*=5.8 Hz, 2H), 5.02–4.97 (*m*, 1H), 4.36–4.16 (*m*, 2H), 2.60 (*t*, *J*=7.4 Hz, 2H), 1.94 (*d*, *J*=1.3 Hz, 3H), 1.76 (quint, *J*=7.3 Hz, 2H), 1.52–1.27 (*m*, 28H), 0.93 (*t*, *J*=6.6 Hz, 3H); ^13^C-NMR (101 MHz, CD_3_OD): *δ* 174.0, 166.6, 152.8, 151.7, 138.7, 137.2 (*d*, *J*=7.6 Hz), 135.9, 129.8, 127.1, 122.5, 112.0, 90.9, 87.2 (*d*, *J*=8.1 Hz), 68.3, 67.8, 35.0, 33.0, 30.8, 30.7, 30.6, 30.5, 30.4, 30.2, 26.0, 23.7, 14.4, 12.5; ^31^P-NMR (162 MHz, CD_3_OD): *δ* −11.14 (*d*, *J*=18.2 Hz), −11.44 (*d*, *J*=19.9 Hz), −23.82 (*t*, *J*=18.6 Hz); IR: 3,209, 3,066, 2,925, 1,757, 1,704, 1,251 cm^−1^; MALDI-MS (*m/z*): [M-H]^−^ calcd. for C_35_H_55_N_2_O_15_P_3_, 835.274; found, 835.398.

### 3′-*O*-Acetylthymidine **20**

The synthesis was carried out as described previously^53^.

TLC (CH_2_Cl_2_/MeOH 9:1): *R*_f_=0.59; ^1^H-NMR (400 MHz, DMSO-*d*_6_): *δ* 11.32 (br.s, 1H), 7.73 (*d*, *J*=1.4 Hz, 1H), 6.17 (dd, *J*=8.7 Hz, *J*=5.9 Hz, 1H), 5.24–5.19 (*m*, 1H), 5.20 (*t*, *J*=5.1 Hz, 1H), 3.99–3.95 (*m*, 1H), 3.62 (dd, *J*=5.3 Hz, *J*=3.5 Hz, 2H), 2.33–2.15 (*m*, 2H), 2.06 (*s*, 3H), 1.78 (*d*, *J*=1.4 Hz, 3H); ^13^C-NMR (101 MHz, DMSO-*d*_6_): *δ* 170.0, 163.7, 150.5, 135.8, 109.7, 84.6, 83.7, 74.7, 61.3, 36.5, 20.8, 12.3. IR: 3,468, 3,181, 1,706, 1,659 cm^−1^; HRMS (*m/z*): [M+Na]^+^ calcd. for C_12_H_16_N_2_O_5_, 307.0901; found, 307.0882.

### Thymidine diphosphate **22** (TDP, tetra-*n*-butylammonium salt)

To a suspension of 1.4 g 3′-*O*-acetylthymidine **20** (1.8 mmol, 1.0 eq.) in 30 ml acetonitrile, 1.3 ml di*iso*propylethlyamine (0.98 g, 7.6 mmol, 1.5 eq.) was added, followed by 1.4 g 5-chlorosaligenylchlorophosphite **8** (6.1 mmol, 1.2 eq.). The reaction mixture was stirred for 3 h and subsequently cooled to 0 °C. By addition of 1.4 ml of a 5.5-M solution of *tert*-butylhydroperoxide in *n*-decane (7.6 mmol, 1.5 eq.) the phosphite was oxidized for 20 min. The solvent was removed in vacuum. The residue was redissolved in CH_2_Cl_2_ and washed with 1 M ammonium acetate solution. The organic phase was dried over Na_2_SO_4_, filtered and the solvent was removed under reduced pressure. The product **21** (quantitative conversion) was used for further steps without purification.

5-chloro-*cyclo*Sal-3′-*O*-acetyl-thymidinemonophosphate **21** (0.51 g; 1.0 mmol, 1.0 eq.) was reacted with 0.89 g mono-(tetra-*n*-butylammonium)-monophosphate (2.6 mmol, 2.5 eq.) in 10 ml DMF. After being stirred for 20 h, the solvent was removed in vacuum and the residue was redissolved in a mixture of methanol/water/tetra-*n*-butylammoniumhydroxide solution (40%) in water (7:3:1 v/v/v). The reaction mixture was stirred for 17 h for deacetylation, followed by removal of the solvent in vacuum. After extraction with water/ethyl acetate, the separated aqueous layer was freeze-dried. The crude product was purified using RP-18 chromatography (water/acetonitrile gradient: 8:1 to 4:1 v/v). Yield: 0.46 g (0.52 mmol, 59%, 2 × Bu_4_N^+^) colourless solid. TLC (*iso*propanol/NH_3_/water 4:1:2.5 v/v/v): *R*_f_=0.19; ^1^H-NMR (300 MHz, D_2_O): *δ* 7.76 (*d*, *J*=1.4 Hz, 1H), 6.32 (dd, *J*=7.6 Hz, *J*=6.4 Hz, 1H), 4.67–4.58 (*m*, 1H), 4.22–4.08 (*m*, 3H), 3.30–2.25 (*m*, 16H), 2.43–2.24 (*m*, 2H), 1.91 (*d*, *J*=1.4 Hz, 3H), 1.74–1.51 (*m*, 16H), 1.35 (sext, *J*=7.4 Hz, 16H), 0.93 (*t*, *J*=7.3 Hz, 24H); ^13^C-NMR (75 MHz, D_2_O): *δ* 166.3, 151.6, 137.3, 111.7, 85.5, 84.9, 71.0, 65.3, 58.1, 38.6, 23.1, 19.1, 12.8, 11.6; ^31^P-NMR (162 MHz, D_2_O): *δ* −10.89 (*d*, *J*=20.0 Hz), −11.53 (*d*, *J*=20.0 Hz); IR: 3,165, 2,960, 2,875, 1,683 cm^−1^; HRMS (ESI^+^, *m/z*): [M+H]^+^ calcd. for C_10_H_16_N_2_O_11_P_2_, 401.016; found, 400.789.

### γ-Bis-(4-nonanoyloxybenzyl)-TTP **3r** (ammonium salt)

General procedure D with 0.11 g TDP **22** (0.13 mmol, 1.0 eq.), 0.17 g **5e** (0.25 mmol, 2.0 eq.), 0.66 ml 0.25 M DCI solution (0.17 mmol, 1.3 eq.), 46 μl 5.5 M solution of *t*-BuOOH in *n*-decane (0.25 mmol, 2.0 eq.) in 0.7 ml acetonitrile. The crude product was purified using automatic RP-18 chromatography (water/acetonitrile gradient). Yield: 95 mg (94 μmol, 74%) colourless solid. UV (HPLC): *λ*_max_=266 nm; HPLC: *t*_R_=16.56 min (method A); ^1^H-NMR (300 MHz, CD_3_OD): *δ* 7.83 (*d*, *J*=1.3 Hz, 1H), 7.42–7.33 (*m*, 4H), 7.03–6.96 (*m*, 4H), 6.28 (dd, *J*=7.6 Hz, *J*=6.0 Hz, 1H), 5.17 (*d*, *J*=8.0 Hz, 4H), 4.65–4.58 (*m*, 1H), 4.30 (ddd, *J*=11.4 Hz, *J*=5.9 Hz, *J*=2.8 Hz, 1H), 4.24–4.14 (*m*, 1H), 4.01–3.90 (*m*, 1H), 2.54 (*t*, *J*=7.4 Hz, 4H), 2.31–2.18 (*m*, 1H), 2.12 (ddd, *J*=13.5 Hz, *J*=6.1 Hz, *J*=3.3 Hz, 1H), 1.89 (*d*, *J*=1.3 Hz, 3H), 1.75–1.51 (*m*, 4H), 1.47–1.21 (*m*, 20H), 0.93 (*t*, *J*=6.7 Hz, 6H); ^13^C-NMR (75 MHz, CD_3_OD): *δ* 173.7, 166.7, 152.4, 152.2, 138.3, 135.2 (*d*, *J*=7.1 Hz), 130.5, 122.8, 112.0, 87.6, 85.8, 72.2, 70.2 (*d*, *J*=5.4 Hz), 67.0, 40.5, 35.0, 33.0, 30.4, 30.3, 30.2, 26.0, 23.7, 14.5, 12.7; ^31^P-NMR (162 MHz, CD_3_OD): *δ* −13.62 (*d*, *J*=22.0 Hz), −15.17 (*d*, *J*=17.8 Hz), −23.67 (*d*, *J*=20.0 Hz); IR: 3,182, 2,924, 1,755, 1,688 cm^−1^; MALDI-MS (*m/z*): [M-H]^−^ calcd. for C_42_H_61_N_2_O_18_P_3_, 973.306; found, 973.491.

### Chemical hydrolysis of Tri*PPP*ro-d4TTP compounds **3a–q** and intermediates **4a,e,j**

Stock solutions (50 mM in DMSO-*d*_6_) of the appropriate compounds were prepared. After dilution of 11 μl with 100 μl Millipore water and 189 μl DMSO-*d*_6_ to 1.9 mM hydrolysis solutions the reaction was started by the addition of 300 μl PBS (50 mM, pH 7.3). The solution was incubated at 37 °C in a thermomixer. An initial aliquot (25 μl) was taken directly and analysed by analytical HPLC at 265–266 nm. Further aliquots were taken for monitoring the kinetic hydrolysis. The exponential decay curves (pseudo-first order) based on absolute integral values were calculated with commercially available software (OriginPro 9.0G) and yielded the half-lives *t*_1/2_(1) and *t*_1/2_(2) of the prodrugs via one determination.

### Enzymatic hydrolysis of Tri*PPP*ro-d4TTP compounds **3a–n** and intermediates **4a,e,j** with PLE

Overall, 20 μl of the appropriate 50 mM DMSO-*d*_6_ stock solution were diluted to 6.0 mM by addition of 83.3 μl DMSO-*d*_6_ as well as 83.3 μl Millipore water. Furthermore, 140 μl of the 6.0 mM solution was diluted with 105 μl DMSO-*d*_6_ and 700 μl 50 mM PBS buffer. The reaction was started by addition of 52.5 μl of PLE in PBS buffer (3 mg ml^−1^) and the mixture was incubated at 37 °C in a thermomixer. At different times, aliquots (125 μl) were taken and treated as follows: (a) for **3a–g**,**l**,**m** and **4a**,**e** the reaction was stopped by addition to 132.5 μl MeOH. The mixture was kept for 5 min on ice followed by centrifugation for 5 min (13,000 r.p.m.). The supernatant was filtered (Chromafil RC-20/15 MS, 0.2 μm) and stored in liquid nitrogen. (b) For **3h**,**i**,**k**,**n** and **4j**, the sample was directly frozen in liquid nitrogen. The solution was defrosted followed by ultrasonication for 10 min. After centrifugation for 5 min, the supernatant was filtered (Chromafil AO-20/3, 0.2 μm) and stored at −196 °C. (c) For **3j**, the mixture was diluted with 70 μl THF (HPLC grade) and frozen in liquid nitrogen followed by defrosting, ultrasonication, centrifugation, filtration and stored as described for (b).

Samples were defrosted and 50–80 μl were subjected to HPLC analysis. The calculation of *t*_1/2_ was performed analogously to that for the chemical hydrolysis studies.

### Enzyme-catalysed hydrolysis of Tri*PPP*ro-d4TTP compounds **3a–n** and intermediates **4a,e,j** in CEM cell extracts

A volume of 10 μl of the appropriate 50 mM DMSO-*d*_6_ stock solution was diluted to 6.0 mM hydrolysis solution by addition of 73.3 μl DMSO-*d*_6_. Seven different samples including 10 μl water and 10 μl hydrolysis solution were prepared. The reaction was started by addition of 50 μl human CEM cell extract and the mixture was incubated at 37 °C for different time periods of hydrolysis. The work-up depended on the particular compound: (a) for **3a–f**,**l**,**m**,**p** and **4a**,**e** the reactions were stopped by addition of 150 μl MeOH. The solution was kept on ice for 5 min followed by centrifugation for 5 min (13,000 r.p.m.). The supernatants were filtered (Chromafil RC-20/15 MS, 0.2 μm) and stored in liquid nitrogen. (b) For **3g–i**,**k**,**n** and **4j**, the samples were directly frozen in liquid nitrogen. The solution was defrosted followed by ultrasonication for 10 min. After centrifugation for 5 min the supernatants were filtered (Chromafil AO-20/3, 0.2 μm) and stored at −196 °C. (c) For **3j** the mixture was diluted with 70 μl THF (HPLC grade) and frozen in liquid nitrogen followed by defrosting, ultrasonication, centrifugation, filtration and stored as described for (b). Samples were defrosted and 50–80 μl were subjected to HPLC analysis. The calculation of *t*_1/2_ was performed analogously to that for the chemical hydrolysis studies.

### Preparation of cell extracts

Human CD_4_^+^ T-lymphocyte CEM cells were grown in RPMI-1640-based cell culture medium to a final density of ∼3·10^6^ cells ml^−1^. Then, cells were centrifuged for 10 min at 1,250 r.p.m. at 4 °C, washed twice with cold PBS and the pellet was resuspended at 10^8^ cells ml^−1^ and sonicated (Hielscher Ultrasound Techn., 100% amplitude, three·times for 10 s) to destroy cell integrity. The resulting cell suspension was then centrifuged at 10,000 r.p.m. to remove cell debris, and the supernatant divided into aliquots before being frozen at −80 °C and used.

### Anti-HIV activity assay

Inhibition of HIV-1(III_B_)- and HIV-2(ROD)-induced cytopathicity in wild-type CEM/0 and TK-deficient CEM/TK^−^ cell cultures was measured in microtitre 96-well plates containing ∼3 × 10^5^ CEM cells ml^−1^ infected with 100 CCID_50_ of HIV per millilitre and containing appropriate dilutions of the test compounds. After 4−5 days of incubation at 37 °C in a CO_2_-controlled humidified atmosphere, CEM giant (syncytium) cell formation was examined microscopically. The EC_50_ (50% effective concentration) was defined as the compound concentration required to inhibit HIV-induced giant cell formation by 50%.

### Primer extension reactions

The used polymerase HIV RT was obtained from Roboklon. The primer and template were purchased from Life Technologies.

Primer sequence:

5′-TTGGATAGGAGGAAGTCCTGGTTGC-3′

Template sequence:

5′-AGACAAACCTATCCTCCTTCAGGACCAACG-3′

The primer extension assays were performed under the following conditions:

The primer was labelled using [γ^32^P]-ATP according to standard techniques. After 5-min incubation at 95 °C in 20 mM Tris-HCl (pH 7.6) and 50 mM NaCl, the hybridization/annealing of the primer to the template strand was achieved by a cooling phase from 95 to 20 °C over 3 h. The final assay solution (20 μl) consists of 2.5 μM dNTPs or hydrolysate, 1 × reaction buffer (50 mM Tris-HCl (pH 8.6), 10 mM MgCl_2_ and 40 mM KCl), 0.02 μM of hybridization and 0.2 units of the enzyme, which was incubated at 37 °C for 10 min. The reaction was stopped by heating up to 80 °C for 3 min. The assays were separated using a denaturating PAGE (15%). The results were visualized by phosphorimaging.

## Additional information

**How to cite this article:** Gollnest, T. *et al*. Lipophilic prodrugs of nucleoside triphosphates as biochemical probes and potential antivirals. *Nat. Commun.* 6:8716 doi: 10.1038/ncomms9716 (2015).

## Supplementary Material

Supplementary Information Supplementary Figures 1-7

## Figures and Tables

**Table 1:** Hydrolysis half-lives of Tri*PPP*ro-d4TTPs **3** and monoesterified d4TTPs **4** in different media.

**Compd**	**R**	**PBS, pH=7.3 (h)**		**PLE (h)**		**CEM (h)**
		***t***_**1/2**_(1)*	***t***_**1/2**_**(2)**†	***t***_**1/2**_(1)*	***t***_**1/2**_**(2)**†	***t***_**1/2**_(1)*
**3a**	CH_3_	18	75	1.9	71	0.050
**3b**	C_2_H_5_	17	150	0.42	33	0.12
**3c**	C_4_H_9_	22	270	0.063	7.7	0.43
**3d**	C_6_H_13_	26	350	0.013	1.6	0.98
**3e**	C_8_H_17_	52	390	0.013	1.6	2.5
**3f**	C_9_H_19_	44	350	0.082	3.0	2.8
**3g**	C_11_H_23_	68	410	0.95	8.3	2.2
**3h**	C_13_H_27_	90	355	30	n.d.‡	4.6
**3i**	C_15_H_31_	73	462	33	n.d.‡	5.3
**3j**	C_17_H_35_	50	583	37	n.d.‡	13
**3k**	C_17_H_33_ (8*Z*)	27	92	42	n.d.‡	4.3
**3l**	OCH_3_	24	200	3.8	177	0.97
**3m**	OC_8_H_17_	82	590	0.12	17	2.6
**3n**	OC_11_H_23_	99	631	44	n.d.‡	3.0
**3o**	NCH_3_(C_9_H_19_NO_2_)	27	n.d.‡	n.d.§	n.d.§	n.d.‡
**3p**	NCH_3_(C_13_H_27_NO_2_)	48	n.d.‡	n.d.§	n.d.§	5.6
**3q**	NCH_3_(C_17_H_35_NO_2_)	48	n.d.‡	n.d.§	n.d.§	n.d.‡
**4a**	CH_3_	n.a.||	95	n.a.||	108	0.040
**4e**	C_8_H_17_	n.a.||	237	n.a.||	1.5	1.8
**4j**	C_17_H_35_	n.a.||	637	n.a.||	57	4.6

^*^Half-lives of **3**.

^†^Half-lives of **4**.

^‡^Not determined.

^§^No substrate for enzyme.

^||^Not available.

**Table 2:** Antiviral activity and cytotoxicity of Tri*PPP*ro-d4TTPs **3** in comparison with the parent nucleoside d4T **1**.

**Compd**	**EC**_**50**_ (μM)*			**CC**_**50**_ **(μM)**†
	**CEM**		**CEM/TK**^−^	**CEM**
	**HIV-1**	**HIV-2**	**HIV-2**	
**3a**	0.43±0.25	0.72±0.16	>10	63±2
**3b**	0.46±0.21	1.16±0.15	>10	57±6
**3c**	0.40±0.00	1.05±0.30	>10	58±3
**3d**	0.36±0.06	0.94±0.16	10±0.00	74±2
**3e**	0.31±0.01	0.62±0.30	2.26±1.03	52±1
**3f**	0.25±0.07	0.33±0.03	0.50±0.14	34±5
**3g**	0.21±0.01	0.27±0.06	0.72±0.16	26±0
**3h**	0.50±0.14	1.10±0.23	1.63±0.52	28±7
**3i**	0.62±0.30	0.66±0.08	0.72±0.16	61±3
**3j**	0.17±0.00	0.31±0.00	0.28±0.04	29±9
**3k**	0.30±0.01	0.47±0.10	0.93±0.47	25±1
**3l**	0.40±0.00	0.92±0.12	>10	16±1
**3m**	0.36±0.06	0.47±0.10	1.26±0.00	51±5
**3n**	0.50±0.14	0.69±0.21	1.26±0.00	41±12
**d4T**	0.33±0.11	0.89±0.00	150±9	79±3

^*^Antiviral activity in CD4^+^ T-lymphocytes: 50% effective concentration; values are the mean±s.d. of *n*=2–3 independent experiments.

^†^Cytotoxicity: 50% cytostatic concentration or compound concentration required to inhibit CD4^+^ T-cell (CEM) proliferation by 50%; values are the mean±s.d. of *n*=2–3 independent experiments.
