# Supplementary material for: Lipophilic prodrugs of nucleoside triphosphates as biochemical probes and potential antivirals
Source: Nat Commun. 2015 Oct 27;6:8716. doi: 10.1038/ncomms9716 (PMC4640093; doi:10.1038/ncomms9716)
Supplement: Supplementary Information — Supplementary Figures 1-7 [file ncomms9716-s1.pdf]

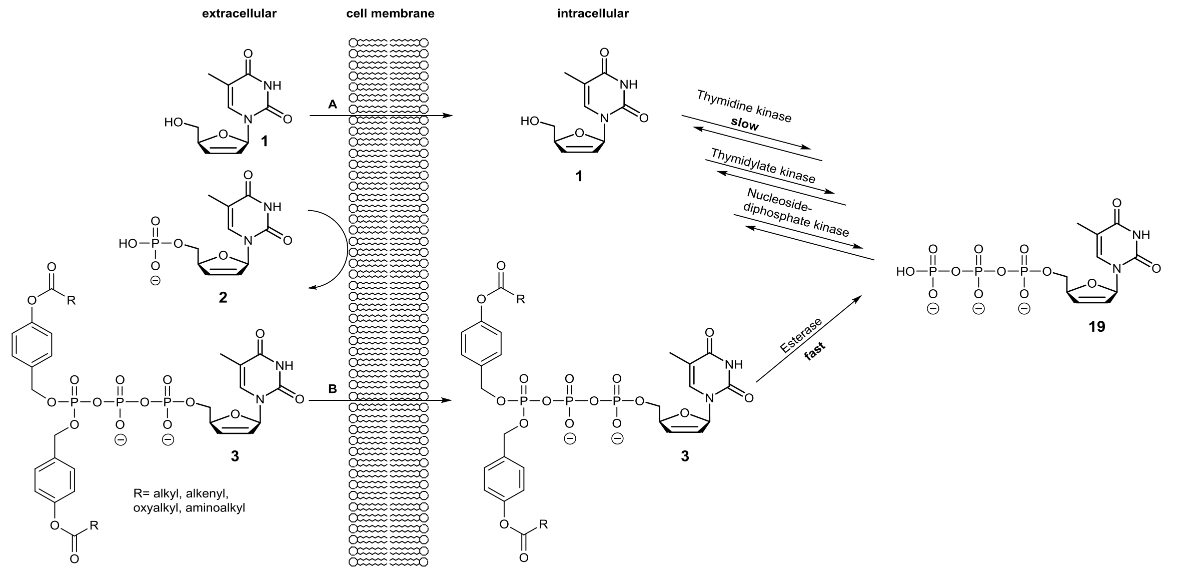


**Supplementary Figure 1** | Cellular uptake, phosphorylation of drug by host cell kinases (A) or uptake and direct release of the biologically active d4TTP **19** from a lipophilic d4TTP prodrug **3** (B).

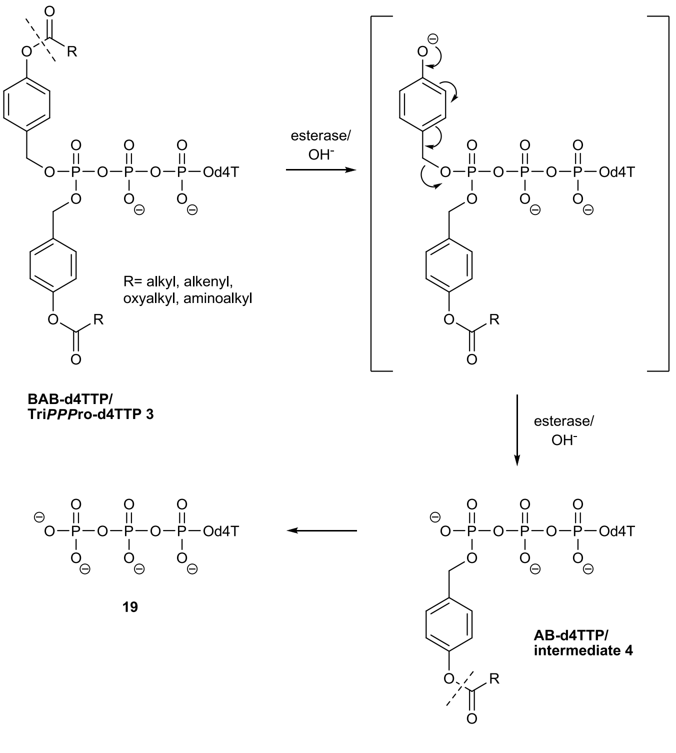


**Supplementary Figure 2** | Release of d4TTP **19** by an enzyme-induced hydrolysis of Tri*PPP*Pro-d4TTP **3**.

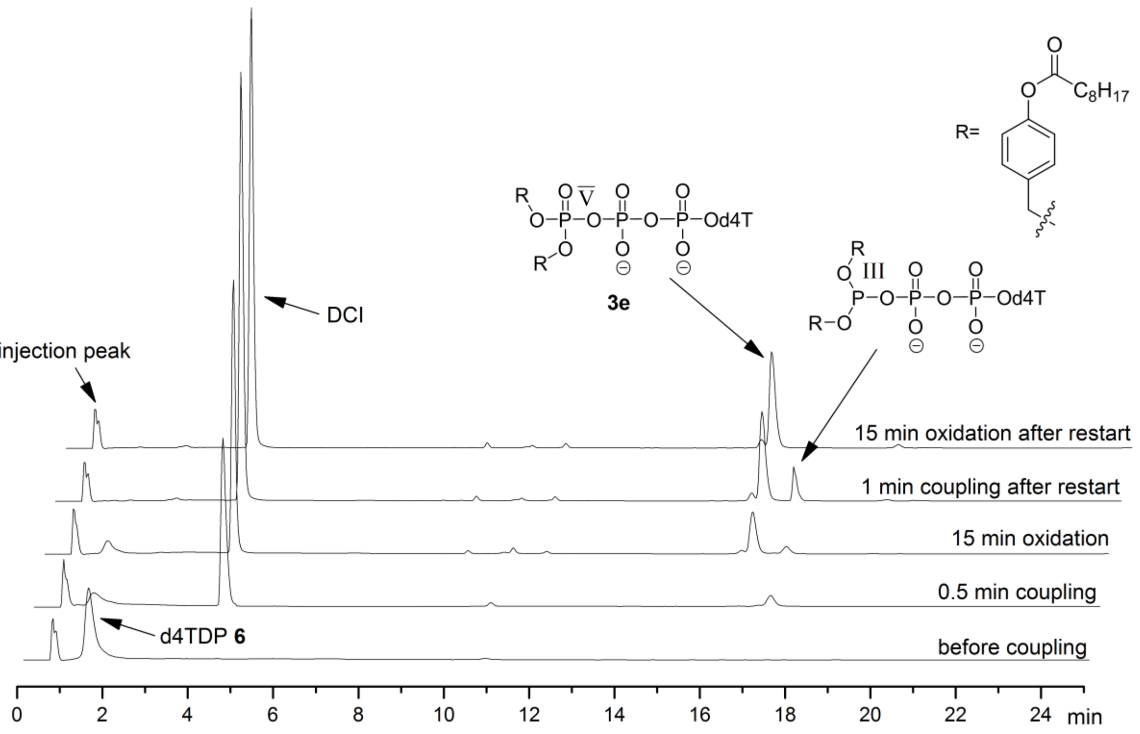


**Supplementary Figure 3** │Reaction monitoring of the synthesis of C8-Tri*PPP*ro-d4TTP **3e** (HPLC-Method B).

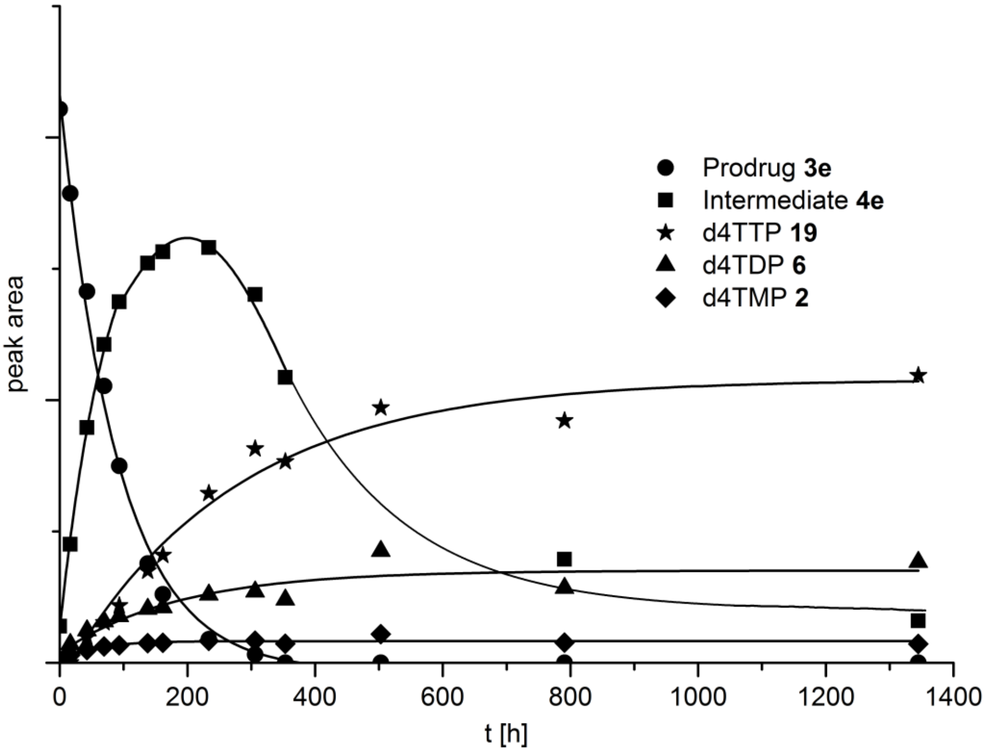


**Supplementary Figure 4** │Process of chemical hydrolysis of C8-Tri*PPP*ro-d4TTP **3e** in PBS (pH 7.3).

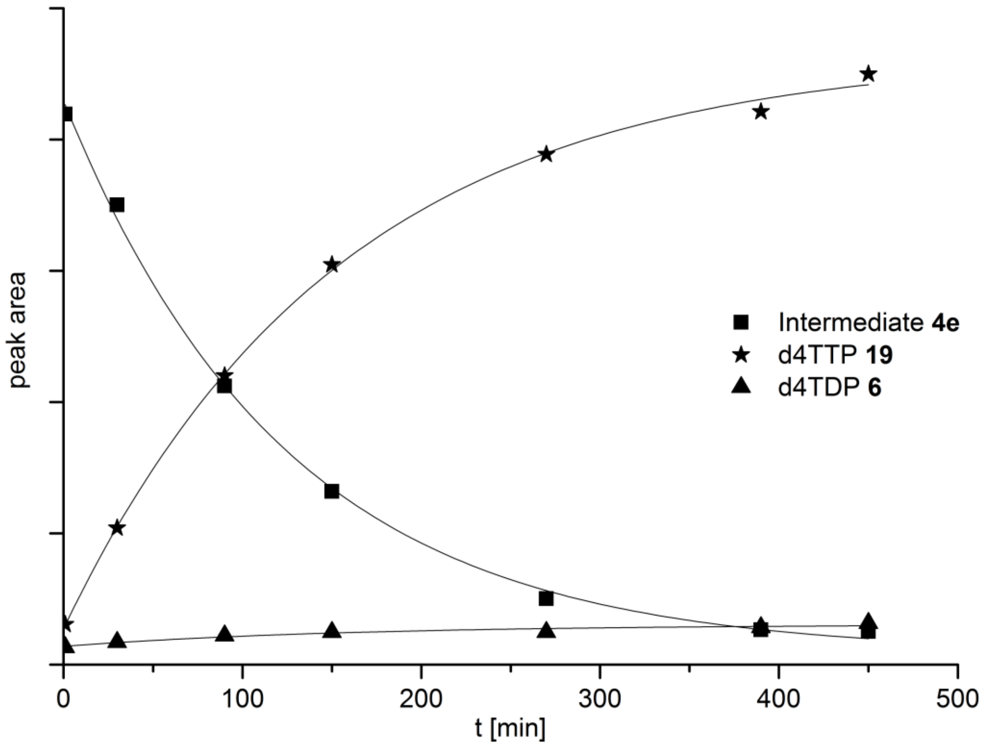


**Supplementary Figure 5** | Enzymatic hydrolysis of mono-esterified d4TTP **4e** with PLE.

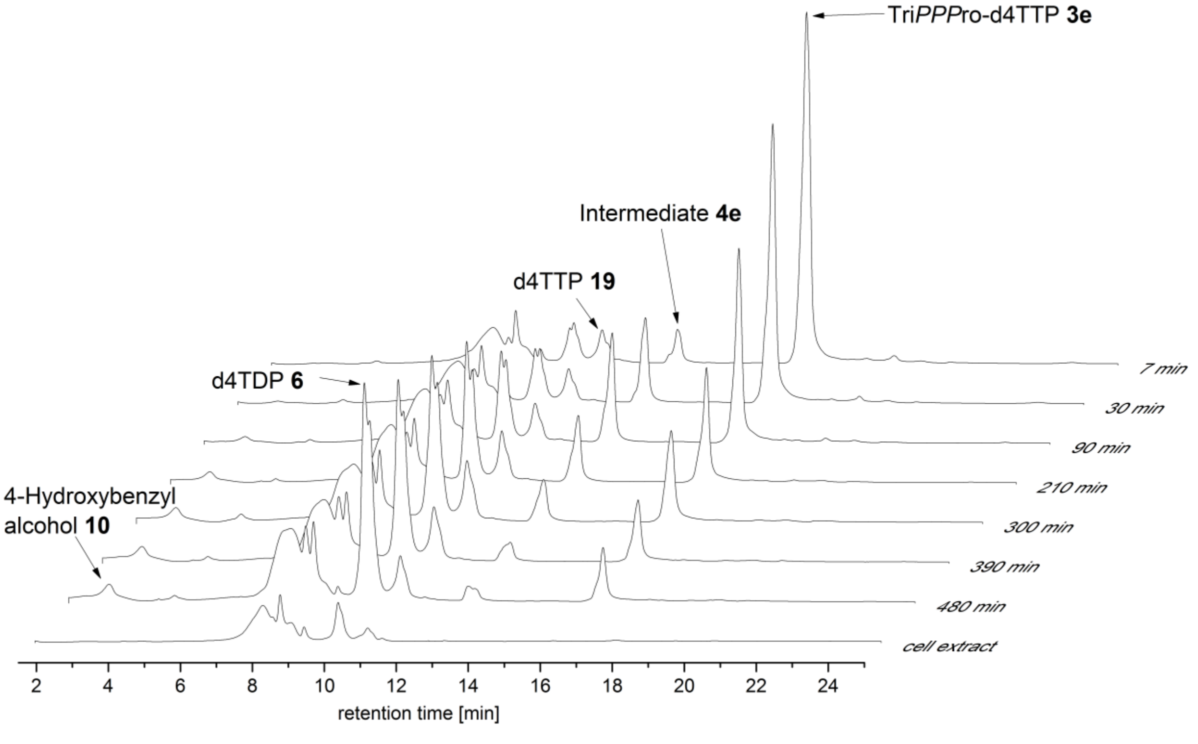


**Supplementary Figure 6** | HPLC profile for Tri*PPP*ro-d4TTP 3e after incubation in CEM cell extracts (HPLC-Method A).

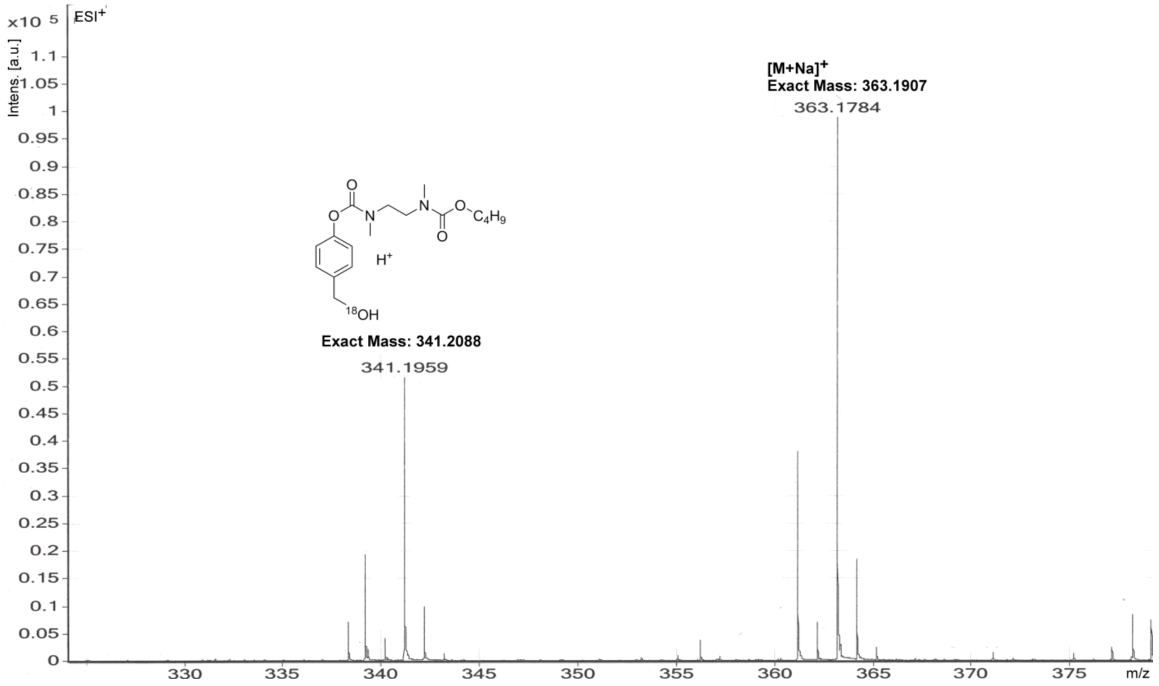


**Supplementary Figure 7** ∣Mass spectra (ESI$^+$) as the result of chemical hydrolysis in PBS buffer of derivative **3o** (performed in $^{18}$O-labeled water).
